# Thioredoxin‐interacting protein (TXNIP) is a substrate of the NEDD4‐like E3 ubiquitin‐protein ligase WWP1 in cellular redox state regulation of acute myeloid leukemia cells

**DOI:** 10.1002/1878-0261.13722

**Published:** 2024-10-04

**Authors:** Sara Giovannini, Yanan Li, Rosalba Pecorari, Claudia Fierro, Claudia Fiorilli, Federica Corigliano, Valeria Moriconi, Ji Zhou, Anna De Antoni, Artem Smirnov, Sara Rinalducci, Anna Maria Timperio, Massimiliano Agostini, Jinping Zhang, Yufang Shi, Eleonora Candi, Gerry Melino, Francesca Bernassola

**Affiliations:** ^1^ Department of Experimental Medicine, TOR University of Rome Tor Vergata Italy; ^2^ The First Affiliated Hospital of Soochow University, Institutes for Translational Medicine, Soochow University Suzhou China; ^3^ Biochemistry Laboratory Istituto Dermopatico Immacolata (IDI‐IRCCS) Rome Italy; ^4^ Institutes of Biology and Medical Sciences, Suzhou Medical College Soochow University Suzhou China; ^5^ IFOM ETS‐The AIRC Institute of Molecular Oncology Milan Italy; ^6^ Department of Ecological and Biological Sciences University of Tuscia Viterbo Italy

**Keywords:** acute myeloid leukemia, HECT E3 ubiquitin ligases, protein ubiquitination, redox homeostasis, ROS

## Abstract

The HECT‐type E3 ubiquitin WWP1 (also known as NEDD4‐like E3 ubiquitin‐protein ligase WWP1) acts as an oncogenic factor in acute myeloid leukemia (AML) cells. *WWP1* overexpression in AML confers a proliferative advantage to leukemic blasts (abnormal immature white blood cells) and counteracts apoptotic cell death and differentiation. In an effort to elucidate the molecular basis of WWP1 oncogenic activities, we identified WWP1 as a previously unknown negative regulator of thioredoxin‐interacting protein (TXNIP)‐mediated reactive oxygen species (ROS) production in AML cells. TXNIP inhibits the disulfide reductase enzymatic activity of thioredoxin (Trx), impairing its antioxidant function and, ultimately, leading to the disruption of cellular redox homeostasis. In addition, TXNIP restricts cell growth and survival by blocking glucose uptake and metabolism. Here, we found that WWP1 directly interacts with TXNIP, thus promoting its ubiquitin‐dependent proteasomal proteolysis. As a result, accumulation of TXNIP in response to WWP1 inactivation in AML blasts reduces Trx activity and increases ROS production, hence inducing cellular oxidative stress. Increased ROS generation in WWP1‐depleted cells culminates in DNA strand breaks and subsequent apoptosis. Coherently with TXNIP stabilization following WWP1 inactivation, we also observed an impairment of both glucose up‐take and consumption. Hence, a contribution to the increased cell death observed in WWP1‐depleted cells also possibly arises from the attenuation of glucose up‐take and glycolytic flux resulting from TXNIP accumulation. Future studies are needed to establish whether TXNIP‐dependent deregulation of redox homeostasis in WWP1‐overexpressing blasts may affect the response of leukemic cells to chemotherapeutic drugs.

## Graphical abstract

Deregulation of *WWP1* expression in acute myeloid leukemia (AML) blasts might accelerate TXNIP proteasomal degradation, thus increasing the ability of thioredoxin to buffer ROS, and ultimately favoring leukemic cell survival. Concomitantly, TXNIP destabilization in *WWP1*‐overexpressing leukemic cells would restrict its capacity to inhibit intracellular glucose transport and consumption, further contributing to the promotion of cancer cell survival and fitness.

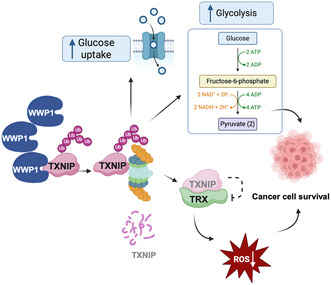

**Abbreviations**
2‐DG: 2‐deoxyglucose
AML: acute myeloid leukemia
ATM: ataxia telangiectasia mutated protein
CHX: cycloheximide
CM‐H2DCFDA: chloromethyl‐2′7′‐dichlorodihydrofluorescein diacetate acetyl ester
Doxy: doxycyclin
DTT: dithiothreitol
E3: E3 protein ubiquitin ligase
ECAR: extracellular acidification rate
EDTA: ethylenediaminetetraacetic acid
glycoPER: glycolytic proton efflux rate
GSH: glutathione
H_2_O_2_
: hydrogen peroxide
HK2: hexokinase 2
LDHA: lactate dehydrogenase A
LDHB: lactate dehydrogenase B
NAC: *N*‐acetyl cysteine
OCR: oxygen consumption rate
PER: proton efflux rate
Prxs: peroxiredoxins
ROS: reactive oxygen species
Rot/AA: rotenone/antimycin A
Trx: thioredoxin
TrxRs: thioredoxin reductases
TXNIP: thioredoxin‐interacting protein
UHPLC: ultrahigh‐performance liquid chromatography
WWP1: WW domain containing E3 ubiquitin protein ligase 1

## 1 Introduction

WW domain containing E3 ubiquitin protein ligase 1 (WWP1) belongs to the C2‐WW‐HECT type sub‐family of E3 protein ubiquitin ligase (E3s), which is composed of additional eight members, including NEDD4, NEDD4‐2, ITCH, SMURF1, SMURF2, WWP2, NEDL1 and NEDL2 [1]. WWP1 contains an N‐terminal protein kinase C (PKC)‐related C2 domain, four tandem WW domains for substrate recruitment, and a C‐terminal catalytic HECT domain for ubiquitin transfer [2]. WWP1 can either functions by its own or require the cooperation with other E3s, the recruitment of adaptors or the occurrence of post‐translational modifications [3, 4]. WWP1 preferentially assembles Lys63 (K63), Lys48 (K48), Lys11 (K11) and Lys27 (K27) polyubiquitin linkages, thus exerting both proteolytic and non‐proteolytic functions [5]. WWP1 is an oncogenic factor implicated in the maintenance of several human cancers [3, 6, 7, 8]. *WWP1* is indeed aberrantly expressed in a variety of human cancers such as prostate, breast, gastric and hepatocellular carcinomas as well as acute myeloid leukemia (AML) [8, 9, 10, 11]. Molecular basis of *WWP1* deregulation include altered transcription, DNA copy number gain, and amplification, whereas somatic and germline mutations are not frequent in cancer cells [12, 13]. Of note, aberrant expression of *WWP1* is often associated with poor prognosis in several cancers [11, 12, 13]. Mechanistically, WWP1 regulates the degradation, the sub‐cellular localization and the activity of protein substrates displaying tumor suppressive functions, such as PTEN, *p27* ^Kip1^, LATS1 and p53 [3, 14, 15, 16], that are of pivotal importance in cancer [17, 18, 19, 20, 21]. It also regulates several pathways crucial for tumor cell proliferation such as the PI3K/AKT, TGF‐β, and Hippo signaling [12]. As a result, WWP1 depletion promotes cell cycle arrest and promotes cancer cell survival. Nevertheless, the molecular basis of the oncogenic activity of WWP1 still remains to be fully elucidated. In particular, evidence linking WWP1 to the regulation of cellular redox state in cancer cells is still lacking.

The thioredoxin‐interacting protein (TXNIP, also termed VDUP1 for vitamin D upregulated protein or TBP2 for thioredoxin‐binding protein) is an endogenous inhibitor of thioredoxin (Trx) [22]. By binding to its redox active catalytic cysteine residues, TXNIP negatively regulates Trx bioavailability and prevents its disulfide reductase function, thereby promoting reactive oxygen species (ROS) accumulation, and, ultimately, leading to the disruption of redox homeostasis [23]. Reduced glutathione (GSH) and the Trx system represent the two major thiol‐dependent antioxidant mechanisms aimed at the removal of ROS, predominantly hydrogen peroxide (H_2_O_2_) and superoxide [24]. The Trx system consists of thioredoxins (Trx1 and Trx2), peroxiredoxins (Prxs), and thioredoxin reductases (TrxRs). Trxs donate electrons to Prx, a thiol‐dependent peroxidase that removes H_2_O_2_, whereas TrxR maintains Trx in a reduced state by using NADPH as a cofactor. TXNIP is mainly a cytoplasmic protein, that, under oxidative stress, can change subcellular localization, including mitochondria, to interact with mitochondrial Trx2. Increased cellular amounts of TXNIP results in oxidative stress, accumulation of DNA damage and increased apoptosis in tumor cells [25, 26]. In addition, TXNIP is a key negative regulator of glucose metabolism [27, 28, 29]. TXNIP can suppress glucose uptake by inhibiting the expression and limiting the localization of glucose transporters to the plasma membrane [30, 31]. In addition, TXNIP negatively regulates glycolysis by downregulating the expression of key glycolytic enzymes [30, 32]. By modulating the antioxidant systems, cellular metabolism, and the remodeling of extracellular matrix, TXNIP exerts tumor suppressive activities in various human cancers such as colorectal, liver, breast, and lung carcinomas and hematologic malignancies, including AML [33, 34, 35, 36]. Repression of *TXNIP* expression is a relevant step in cancer cell transformation. Several studies have indeed demonstrated downregulation of *TXNIP* expression, frequently associated with disease progression, in human tumors [34]. In AML cells, *TXNIP* expression can be repressed by two epigenetic regulatory mechanisms, histone deacetylation and trimethylation of histone H3 on Lys27 (H3K27me3) [37]. Coherently, reducing H3K27me3 levels results in *TXNIP* mRNA upregulation that ultimately leads to ROS generation and subsequent apoptotic cell death of AML blasts.

## 2 Materials and methods

### 2.1 Cell culture, treatments

OCI‐AML3 cells (RRID: CVCL_1844) were grown in RPMI medium (Thermo Fisher Scientific, Waltham, MA, USA). HEK293T cells (RRID: CVCL_0063) were cultured in Dulbecco's‐modified Eagle's medium (DMEM) (Thermo Fisher Scientific). Both the media were supplemented with 10% fetal bovine serum (FBS, Thermo Fisher Scientific) and contained penicillin (100 U·mL^−1^, Thermo Fisher Scientific) and streptomycin (100 μg·mL^−1^, Thermo Fisher Scientific). The NB4/Tet‐On/shWWP1 cell lines were generated by lentiviral transduction of the NB4 cell line (RRID: CVCL_0005) with the pRSIT16‐U6Tet‐sh‐CMV‐TetR‐2A‐RFP‐Puro vector (Cellecta, Mountain View, CA, USA) for conditional expression of shRNAs against WWP1. The sequences of the WWP1 shRNAs were the following: ATTGCTTATGAACGCGGCTTT (shRNA#1), ATATAAGCGCCTCCTCAAGTC (shRNA #2). Following lentiviral transduction, RFP^+^ cells were isolated through fluorescence‐activated cell sorting. Cells were cultured in RPMI medium supplemented with 10% TET‐free FBS, penicillin and streptomycin. All cell lines were grown at 37 °C in a 5% CO_2_ humidified atmosphere. Doxycyclin (doxy; Sigma, St. Louis, MO, USA) was used at the concentration of 0.5 μg·mL^−1^ for 48 h to induce WWP1 silencing. *N*‐acetyl cysteine (NAC, 10 mm; Sigma) was added to cell medium for 48 h. Cells were exposed to cycloheximide (CHX; 10 μg·mL^−1^; Sigma) for the indicated time. MG132 (Boston Biochem, Cambridge, MA, USA) was added to the medium 4 h before cell harvesting.

All cell lines were provided by ATCC. They have been authenticated in the past 3 years by DNA isolation from cell pellet and subsequent PCR‐single‐locus‐technology. The following independent PCR‐systems D8S1179, D21S11, D7S820, CSF1PO, D3S1358, TH01, D13S317, D16S539, D2S1338, AMEL, D5S818, FGA, D19S433, vWA, TPOX and D18S51 were applied. All experiments were performed with mycoplasma‐free cells.

### 2.2 Metabolite extraction and UHPLC–MS analysis

Metabolite extraction and ultra‐performance liquid chromatography‐mass spectrometry (UHPLC–MS) analysis were performed as previously described [38]. After 48 h incubation with doxy and PBS washing, NB4/Tet‐On/shWWP1 cells were harvested and samples were lysed by adding 0.15 mL of ice‐cold ultra‐pure water (18 MΩ). The tubes were plunged into dry ice on a circulating bath at −25 °C for 30 s and afterwards placed into a water bath at 37 °C for 30 s. To each tube, 0.6 mL of cold methanol and 0.45 mL of cold chloroform were added. The tubes were mixed every 5 min for 30 min. The solutions were then centrifuged for 15 min at 15 000 ***g*** before being transferred to −20 °C for 2–8 h. The tubes were then centrifuged at 10 000 ***g*** for 10 min at 4 °C, and the collected supernatants were dried. Dried samples were re‐suspended in 0.1 mL of water containing 5% formic acid, and then transferred to glass autosampler vials for LC/MS analysis. The extracted supernatant samples (20 μL) were injected into an ultrahigh‐performance liquid chromatography (UHPLC) system (Ultimate 3000, Thermo, Thermo Fisher Scientific) and run in positive mode. A reprosil C18 column (2.0 × 150 mm, 2.5 μm‐DrMaisch, Ammerbuch‐Entringen, Germany) was used for metabolite separation. For positive ion mode (+) MS analyses, a 0–100% linear gradient of solvent A (ddH_2_O, 0.1% formic acid) to solvent B (acetonitrile, 0.1% formic acid) was used for 20 min, returning to 100% A in 2 min and holding solvent A for a 1‐min posttime hold. Chromatographic separations were performed at 30 °C at a flow rate of 0.2 mL·min^−1^. The UHPLC system was coupled with a Q‐Exactive mass spectrometer (Thermo) scanning in full MS mode (2 μ scans) at a resolution of 70 000 in the 67 to 1000 m/z range. Calibration was performed against positive or negative ion mode calibration mixtures (Pierce, Thermo Fisher, Rockford, IL, USA) to ensure error of the intact mass within the sub‐ppm range. Metabolites of interest were statistically analyzed and graphed with graphpad prism 5.01 (Graphpad Software Inc., La Jolla, CA, USA). Data are mean values of five replicates ± SD.

### 2.3 Detection of intracellular ROS

To measure ROS production, cells were labeled with 1 μm CM‐H2DCFDA (5‐(and‐6)‐chloromethyl‐2′7′‐dichlorodihydrofluorescein diacetate acetyl ester (Life Technologies, Carlsbad, CA, USA) for 20 min at 37 °C and MITOSOX (Thermofisher M36008) for 15 min at 37 °C at the concentration of 1 μm). After washing with PBS, cells were resuspended in PBS and ROS generation was assessed by flow cytometry (CytoFLEX cytometer; excitation, 488 nm; emission, 515–545 nm). A total of 10 000 events were evaluated.

### 2.4 JC‐1 staining

For the detection of mitochondrial potential, cells were collected, washed two times with PBS and stained with JC‐1 probe (Thermofisher M34152) for 15 min at 37 °C at the concentration of 2 μm. Flow cytometric data were acquired on a CytoFLEX cytometer (excitation, 537 nm; emission, 618 nm). A total of 10 000 events were evaluated.

### 2.5 Detection of DNA strand breaks by comet assay

Forty‐eight hours after doxy and/or NAC treatment cells were re‐suspended in ice‐cold PBS at a concentration of 10^5^ cells·mL^−1^, embedded in low melting agarose at a ratio of 1 : 10 and spread on Comet slides (Trevigen, Gaithersburg, MD, USA). After ON incubation at 4 °C in Lysis Solution (Comet assay Trevigen kit), the slides were immersed in pre‐chilled Alkaline Unwinding Solution (300 mm NaOH, 1 mm EDTA, pH > 13) for 1 h. Subsequent electrophoresis occurred in Alkaline solution at 21 V for 30 min at 4 °C. Slides were afterwards washed twice in distilled H_2_O, immersed in 70% ethanol for 5 min and dried at 37 °C. They were then stained with SYBR Green for 30 min. Images were taken with a fluorescence microscope (Leica DM6 B, Wetzlar, Germany) and 100 cells were analyzed with imagej in three independent experiments. The analysis was reported in a bar graph (prism), showing the average of three independent assays with standard deviation and significance.

### 2.6 Cell viability assay

Cells were seeded in white 96‐well plates at different time points of DMSO or 0.5 μg·mL^−1^ doxy for the indicated times. The CellTiter‐Glo® (Promega, Madison, WI, USA) reagent was added, shaken for 5 min in the dark, and luminescence signals produced were measured using microplate reader (GloMax, Promega). The viability of each well was calculated by normalizing the luminescent signal to the average signal from time point 0. The data result from three independent experiments with standard deviation and significance.

### 2.7 Apoptosis analysis by flow cytometry

Cells were collected and fixed with ice‐cold MetOH : Acetone (4 : 1) for 30 min and then incubated with RNAse and Propidium iodide (PI; 50 mg·mL^−1^) at room temperature for 15 min in the dark. Flow cytometric data were acquired on a CytoFLEX cytometer (excitation, 537 nm; emission, 618 nm). A total of 10 000 events were evaluated. Cells (1 × 10^6^·mL^−1^) were also stained using the FITC Annexin V Apoptosis Detection Kit and DAPI according to the manufacturer's instructions (Becton Dickinson, Franklin Lakes, NJ, USA). Stained cells were analyzed using the CytoFLEX cytometer (excitation, 485 and 359 nm; emission, 535 and 457 nm). A total of 10 000 events were evaluated.

### 2.8 Protein extraction and western blot

Protein extracts were prepared with Triton‐NaCl extraction buffer (250 mm NaCl, 50 mm NaF, 1 mm ethylenediaminetetraacetic acid (EDTA), 50 mm Tris–HCl, pH 7.5, 0.5% Triton‐X‐100), supplemented with 1 mm dithiothreitol (DTT), protease and phosphatase inhibitors (100 μm orthovanadate, 100 μm phenylmethylsulfonyl fluoride and Protease inhibitor cocktail (Roche, Basel, Switzerland)). Proteins were separated on polyacrylamide gels and then transferred on a polyvinylidene fluoride transfer membrane (Immobilon, Millipore, Burlington, MA, USA), pre‐activated in 100% MeOH, in chilled Transfer Buffer (25 mm Tris, 192 mm glycine, 20% MeOH) for 2 h at 350 mA, or for 10 min with the Trans‐Blot Turbo Transfer System (Bio‐Rad, Hercules, CA, USA). The membranes were incubated at RT in blocking buffer (EveryBlot, Bio‐Rad) for 5–10 min, or in 5% non‐fat dry milk/BSA for 1 h, depending on the subsequently used antibody, in TBS‐T (20 mm Tris–HCl, pH 7.4, 150 mm NaCl and 0.1% Tween‐20). Incubation with primary antibodies occurred for 2 h at RT or overnight at 4 °C. After three TBS‐T washings, the membranes were incubated with secondary antibodies for 1 h at RT. The following antibodies were used: anti‐WWP1 antibody (Abnova, Taipei, Taiwan), anti‐TXNIP antibody (Cell Signaling, Danvers, MA, USA), anti‐FLAG M2 (Sigma), anti‐BRCA1 (Santa Cruz Biotechnology, Dallas, TX, USA), anti‐γH2AX (ser139) (Millipore 05‐636), anti‐P‐ATM (ser1981) (Cell Signaling), anti‐ATM (Cell Signaling 2873), anti‐PARP (Cell Signaling 9542), anti‐HIF1α (Bethyl Laboratories, Montgomery, TX, USA), anti‐Myc (Cell Signaling, clone 9b11), anti‐HA (Biolegend, San Diego, CA, USA), anti‐β‐actin (Sigma) and anti‐GAPDH (Sigma) antibodies. HRP‐conjugated secondary goat anti‐mouse and anti‐rabbit antibodies (Bio‐Rad), as well as HRP‐conjugated secondary mouse anti‐rabbit or goat anti‐mouse light chain antibodies (Millipore) were used at a dilution of 1 : 5000. After three washes in TBS‐T, the membranes were incubated with Western Lightning Plus Chemiluminescent Substrate (Perkin Elmer, Waltham, MA, USA) and scanned at the UVITEC (Cambridge) [39]. Densitometric analysis was done using imagej software.

### 2.9 Cloning and cells transfections

The human TXNIP transcript variant 1 (NM_006472.6) was PCR amplified. The TXNIP cDNA was cloned into a pcDNA 3.1‐MYC vector using Kpn1 and EcoRI restriction enzymes. The overexpression of FLAG‐tagged wild type (WT) and catalytic mutant (CA) WWP1 proteins was obtained by transfecting HEK293T cells with Effectene (Qiagen, Hilden, Germany), according to the manufacturer's instructions. The overexpression of pcDNA 3.1 FLAG‐tagged WT WWP1 FLAG‐tagged C890A WWP1, pcDNA 3.1 MYC‐tagged TXNIP, pcDNA 3.1 HA‐tagged wild‐type and mutant ubiquitins was obtained by transfecting HEK293T cells with Lipofectamine 3000 (Invitrogen, Waltham, MA, USA), according to the manufacturer's instructions.

The WWP1 and TXNIP siRNAs transfection was carried out with RNAimax (Thermofisher) as manufacturer instruction for 48 h at the concentration of 20 nm. The sequences of the WWP1 siRNAs were the following: CCUAUUAUGUGGAUCAUAA (siWWP1 #1) GUGGAAGGUUGCAGUUACA (siWWP1 #2). The sequences of the TXNIP siRNAs were the following: GACCAUUAACCGUCGUCUAGU (siTXNIP #1) UUCUCGGUUAAAUUGUUUGAU (siTXNIP #2).

### 2.10 Bioinformatic analysis

The WWP1 predicted interactome was obtained using the online database STRING (https://string‐db.org/). The interactors were selected by experimental determined interactions with a minimum required interaction score of 0.4. All the interactor scores are shown in Table S1. The gene ontology analysis of the WWP1 interactors were obtained using the online software david (https://david.ncifcrf.gov/).

### 2.11 Co‐immunoprecipitation assays

For immunoprecipitation, cell lysates were prepared in Triton‐NaCl extraction buffer. One milligram of total protein extracts was resuspended in 500 μL of the specific buffer and incubated for 4–6 h at 4 °C with Protein A sepharose beads (4 Fast flow, GE HealthCare Technologies, Inc. Chicago, IL, USA 17528001) for the pre‐cleaning step. After centrifugation, the beads‐unbound lysates of Hek293T cells expressing FLAG‐tagged WWP1 protein were incubated O.N. with EZview Red anti‐FLAG M2 affinity resin beads (Sigma, F2426). NB4 and OCI‐AML3 cell lysates were incubated with anti‐TXNIP antibody (Cell Signaling 14715) or IgG Rabbit isotype control antibody (DA1E, Cell Signaling 3900) ON at 4 °C. Afterwards, the samples were incubated with protein A beads for 3 h at 4 °C. Finally, after 5–7 washings of 10–20 min each, proteins were eluted with loading buffer and incubated for 5 min at 98 °C. After centrifugation, the supernatant was loaded onto a polyacrylamide gel for electrophoresis analysis.

### 2.12 *In vitro* pull‐down assays

Recombinant human WWP1 protein (300 ng; Abcam, Cambridge, UK) was incubated with recombinant TXNIP protein (300 ng; My Biosource, San Diego, CA, USA) over‐night at 4 °C in binding buffer (50 mm Tris–HCl, pH 7.5, 150 mm NaCl, 0.1% Nonidet NP‐40, EDTA 1 mm). Concurrently, anti‐WWP1 antibody (Novus NBP1‐49713, St. Louis, MO, USA) was incubated with Protein A beads in binding buffer as well. After centrifugation of the beads, the proteins were added to the antibody‐beads complexes and left for 3 h on a rotator at 4 °C. After washing, the loading buffer was added and samples were boiled, centrifuged and the supernatant was used for gel electrophoresis.

### 2.13 *In vivo* ubiquitination assays

Forty‐eight hours posttransfection cells were treated with 10 μm MG132 for 4 h and then harvested. To inhibit ERK kinase activity, cells were treated with 250 nm SCH772984 (DBA‐HY‐50846‐5 mg) for 24 h. Whole‐cell extracts were obtained by lysing the cells with RIPA buffer (50 mm Tris‐cl pH 7.4; 150 mm NaCl; 1% NP40; 0.25% Na‐deoxycholate;1 mm DTT), supplemented with 500 mm NEM (*N*‐ethylmaleimide) and Complete™ Protease Inhibitor Cocktail (Roche). Two milligram of the extract were incubated O.N. at 4 °C with Protein G sepharose beads (4 Fast flow, GE HealthCare Technologies 17528001) for pre‐cleaning, together with Protein G sepharose beads and anti MYC‐Tag primary antibody. Subsequently to three washes in RIPA buffer, the beads conjugated with MYC‐Tag antibody were incubated 3 h with the protein lysates. Finally, after three washes, proteins were eluted with loading buffer and incubated for 5 min at 98 °C. After centrifugation, the supernatant was loaded onto a polyacrylamide gel for electrophoresis analysis.

### 2.14 *In vitro* ubiquitination assay

Purified recombinant TXNIP protein (My Biosource) was incubated with active recombinant WWP1 FLAG‐tagged protein (Sigma SRP0229) in 1 : 1 ratio (100 nm) in a protein buffer (50 mm Tris–HCl, pH 7.5, 200 mm NaCl) for 20 min at 30 °C. After the addition of 4 mm ATP, 10 mm MgCl_2_, 20 mm Tris, pH 7.5, 50 μm Ubiquitin (UBPBio, Dallas, TX, USA), 1 μm UbcH7 E2 (R&D System, Minneapolis, MN, USA) and 100 nm UBE1 (R&D System) the samples were left for 2 h at 30 °C. Finally, samples were incubated at 98 °C for 5 min with loading buffer, prior to gel electrophoresis.

### 2.15 Thioredoxin activity measurement

Cells were lysed with lysis buffer (20 mm HEPES (pH = 7.6), 100 mm KCl, 300 mm NaCl, 10 mm EDTA, 0.1% Nonidet P‐40, plus Protease inhibitor cocktail) on ice for 15 min. The protein concentration of cell extracts was measured as demonstrated before. Cell extracts (40 μg) were preincubated at 37 °C for 20 min with 2 μL of DTT activation buffer (50 mm HEPES (pH 7.6), 1 mm EDTA, 1 mg·mL^−1^ BSA, and 2 mm DTT) in a total volume of 35 μL to reduce TRX. Then, 20 μL of reaction mixture (1 mL mixture include 200 μL of 1 m HEPES (pH = 7.6), 16 μL of 0.5 m EDTA, 40 μL of NADPH (40 mg·mL^−1^), and 500 μL of insulin (10 mg·mL^−1^)) was added. The reaction began with the addition of 4 μL of human recombinant TXNRD1 (0.5 mg·mL^−1^) and then the samples were incubation at 37 °C for 20 min. Finally, the reaction was stopped by the addition of 250 μL of stop solution (6 m guanidine hydrochloride and 0.4 mg·mL^−1^ 5,5‐dithiobis‐(2‐nitrobenzoic acid) in 0.2 m Tris–HCl) and absorbance at 412 nm was measured.

### 2.16 RNA extraction, cDNA, RT‐qPCR

RNA extraction and RT‐qPCR were performed as previously described [40]. In details, total RNA was isolated using the RNeasy mini kit (Qiagen) following the manufacturer's protocol. RNA was quantified using a NanoDrop spectrophotometer (Thermo Scientific). For mRNA expression level analysis, total RNA was reverse transcribed by using SensiFAST cDNA Synthesis Kit (Bioline, London, UK) according to manufacturer's instructions. Real Time‐qPCR was performed using the SensiFAST™ Probe Lo‐ROX Kit (Meridian Bioscience, Cincinnati, OH, USA) in an Applied Biosystems Q3 or Q5 Real‐Time PCR System (Thermo Fisher Scientific). The relative expression of each gene was defined from the threshold cycle (*C*_t_) and calculated using the $2^{-\Delta \Delta C_{t}}$ method. The human (Tata‐Box Binding Protein) TBP was used as a housekeeping gene for normalization. The primers sequences are listed in Table S2.

### 2.17 Glucose uptake

Glucose uptake was assessed with a bioluminescent assay kit (Promega) according to manufacturer's instructions. Briefly, after 48 h incubation with doxy, 30 000 untreated control and treated cells were harvested and washed twice with PBS. 2‐deoxyglucose (2‐DG, 1 mm) was added for 10 min at room temperature. The reaction was stopped by adding 25 μL of Stop Buffer and 25 μL of Neutralization Buffer. Finally, 100 μL 2‐deoxyglucose‐6‐phosphate Detection Reagent was added and, after 30 min of incubation, luminescence intensity (Relative light unit, RLU) was measured.

### 2.18 Glycolytic rate analysis

A Seahorse XFe24 analyzer (Agilent Technologies, CA, USA) was used to measure extracellular acidification rate (ECAR) and oxygen consumption rate (OCR), using the XF Glycolytic Rate Assay Kit (Agilent Technology, Santa Clara, CA, USA) [41]. All parameters were corrected for non‐mitochondrial respiration and background signal from the instrument after the injection of 0.5 μm Rotenone/antimycin A (Rot/AA). The residual acidification rate was detected after the addition of 50 mm 2‐DG to inhibit glycolysis. Rot/AA addition allows calculation of the mitochondrial respiration contribution to the rate of proton efflux. Subtraction of the mitochondrial proton efflux rate from the total proton efflux rate provides the glycolytic proton efflux rate (glycoPER) [42]. For the experiments, cells were seeded in poly‐l‐lysine‐coated Seahorse XFe24 96‐well plates at a density of 75 000 and 100 000 cells per well for NB4/Tet‐On/shWWP1 and OCI‐AML3, respectively, in glucose‐free RPMI medium supplemented with 10 mm glucose, 2 mm glutamine, 1 mm sodium pyruvate, and then centrifuged at 200 ***g*** for 1 min. OCR was measured under the same conditions to assess mitochondrial basal respiration. Three measurements were carried out after the addition of each compound with 4 min mixing intervals, followed by 3 min measuring periods. Collected data were analyzed using the seahorse wave 2.6 software (Agilent Technologies) and normalized to total cell counts determined by Incucyte cell confluency analyses.

### 2.19 ATP rate assay

A Seahorse XFe24 analyzer (Agilent Technologies) was used to measure ATP production rate, using the XF Real‐time ATP Rate Assay Kit (Agilent Technology). All parameters were corrected for background signal from the instrument after the serial injection of 1.5 μm oligomycin and 0.5 μm Rot/AA. 100 000 cells per well were seeded in poly‐l‐lysine‐coated Seahorse XFe24 96‐well plates in glucose‐free RPMI medium supplemented with 10 mm glucose, 2 mm glutamine, 1 mm sodium pyruvate, and then centrifuged at 200 ***g*** for 1 min. Addition of oligomycin was performed to inhibit mitochondrial ATP synthesis, resulting in a decrease in OCR, and allowing the quantification of mitoATP Production Rate. ECAR data combined with the buffer factor of the RPMI assay medium provides the calculation of total Proton Efflux Rate (PER). Complete inhibition of mitochondrial respiration by Rot/AA addition enables measurement of mitochondrial‐associated acidification, which then combined with PER data permits calculation of the glycoATP Production Rate [43]. Three measurements were conducted after the addition of each compound.

### 2.20 Statistical analysis

Data analysis was conducted by using the software graphpad prism. Data are expressed as mean ± SD, and *P*‐values were calculated by unpaired Student's *t*‐test or ANOVA. *P* < 0.05 was used to identify statistically significant differences.

## 3 Results

### 3.1 Inactivation of WWP1 expression elevates ROS levels

**Fig. 1:**
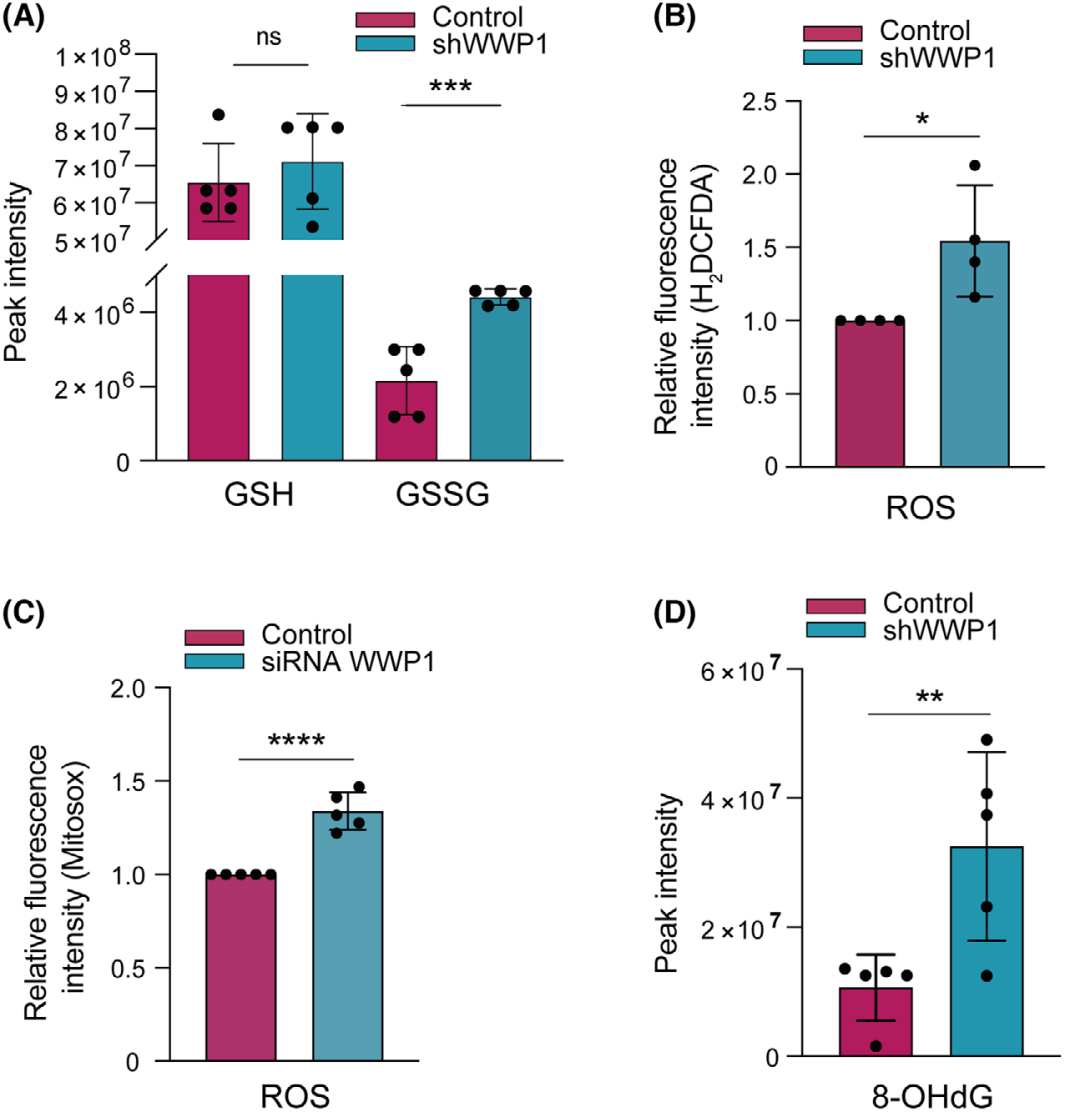
Inducible inactivation of WWP1 expression leads to oxidative stress in AML cells. (A) Glutathione measurements by LC/MS (liquid chromatography‐mass spectrometry) in AML (acute myeloid leukemia) NB4 cells following inducible shRNA‐mediated gene silencing of WWP1. The tetracycline (Tet)‐On‐inducible NB4 cells (hereinafter referred to as NB4/Tet‐On/shWWP1 cells) were either left untreated or incubated with 0.5 μg·mL^−1^ doxy for 48 h to achieve shWWP1 expression. The relative quantification of GSH (reduced glutathione) and GSSG (oxidized glutathione) was based on peak intensity and expressed as the mean ± SD of five independent biological replicates. Statistically significant differences were calculated by unpaired Student's *t*‐test. (B) Detection of ROS (reactive oxygen species) in control and WWP1‐depleted NB4 cells by the H_2_DCFDA assay. NB4/Tet‐On/shWWP1 cells were treated with 0.5 μg·mL^−1^ doxy for 48 h before incubation with 1 μm H_2_DCFDA. ROS levels are expressed as fold increase in fluorescence intensity. Values represent the mean ± SD from four independent experiments. (C) MitoSOX‐based flow cytometric detection of mitochondrial ROS production in control and WWP1‐depleted OCI‐AML3 cells. Cells were transfected with a non‐targeting control siRNA or with a pool of two individual siRNAs: siWWP1 #1 and siWWP1 #2 for 48 h. Values show the mean ± SD of five independent experiments. (D) Levels of oxidative DNA damage assessed by LC/MS‐based quantification of 8‐hydroxy‐2′deoxyguanosine (8‐OHdG) in control and WWP1‐depleted NB4 cells. The relative quantification of 8‐OHdG relied on peak intensity and was expressed as the mean ± SD of five replicates. Statistically significant differences were calculated by unpaired Student's *t*‐test. *P < 0.05, **P < 0.01, ***P < 0.001, ****P < 0.0001.

To better elucidate the molecular mechanisms underlying the oncogenic activity of WWP1, and explore a possible link of WWP1 with oxidative stress, glutathione levels were measured in control and WWP1‐depleted NB4 AML cells by means of LC–MS. In particular, we observed a shifted ratio of the reduced form of glutathione (GSH) versus its oxidized form (GSSG) (Fig. 1A). The ratio GSH/GSSG was indeed 30 and 16 in control and shWWP1 cells, respectively. Since the GSH/GSSG ratio is a dynamic indicator of cellular oxidative stress, we sought to measure the ROS content following WWP1 inactivation. By exploiting the fluorogenic probe 2′–7′ dichlorofluorescin diacetate (H_2_DCFDA) that is mainly oxidized by H_2_O_2_, we found that WWP1‐depleted AML cells indeed contain higher levels ROS relatively to control blasts (Fig. 1B and Fig. S1A,B). We also found WWP1‐depleted leukemic cells display higher levels of mitochondrial ROS, as measured by the superoxide indicator MitoSOX (Fig. 1C), suggesting that WWP1‐mediated regulation of TXNIP may influence the activity of both cytoplasmic/nuclear Thioredoxin1 and mitochondrial Thioredoxin2. Assessment of mitochondrial depolarization revealed a drop of the mitochondrial membrane potential in WWP1‐depleted AML cells that was coherent with the increase of mitochondrial ROS (Fig. S1C). Because the steady state level of ROS can be easily buffered, besides measuring ROS concentrations, it can be also useful to test downstream oxidation products. Upon WWP1 silencing, we observed enhanced oxidative stress‐induced DNA damage assessed as 8‐hydroxy‐2′‐deoxyguanosine (8‐OHdG) generation (Fig. 1D).

### 3.2 WWP1 depletion increases endogenous DNA damage through ROS generation

**Fig. 2:**
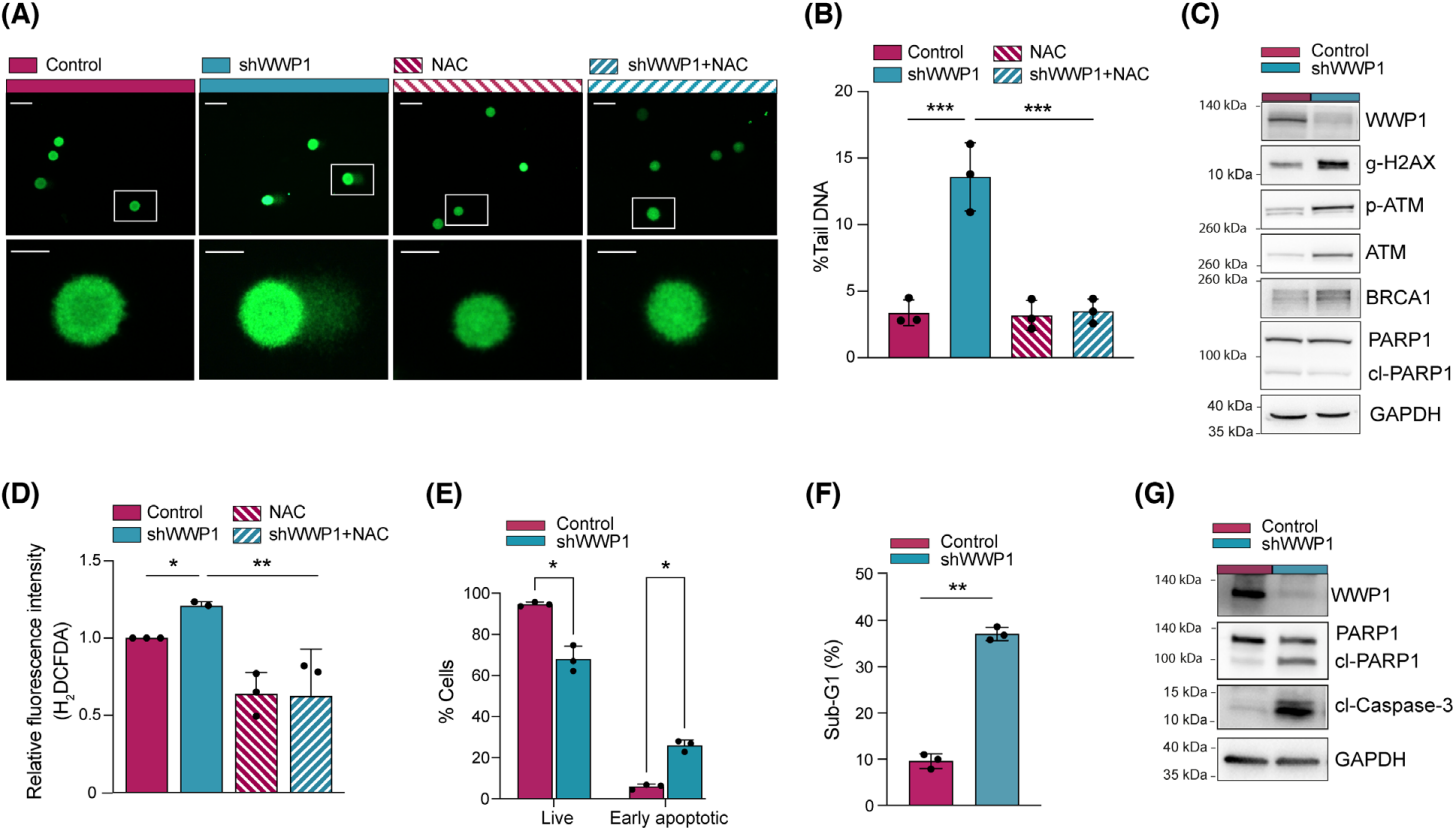
WWP1‐depleted leukemic cells display enhanced levels of ROS‐induced DNA damage. (A, B) DNA strand breaks measurement in untreated control NB4/Tet‐On/shWWP1 cells and in cells incubated with 0.5 μg·mL^−1^ doxy for 48 h. Cells were also treated with 10 mm NAC for 48 h. Representative fluorescence images Representative fluorescence images (scale bar = 50 and 200 μm in upper and lower images, respectively) (A) and quantification (B), in percentage, of tail DNA. The parameter tail DNA% was measured with imagej from 100 randomly selected cells for each condition. Values represent the mean ± SD from three independent experiments. Statistical significance was calculated using one‐way ANOVA test. (C) Representative western blot analysis of γ‐H2AX, ATM, phosphorylated ATM and BRCA1 proteins in control and WWP1‐depleted NB4 cells. NB4/Tet‐On/shWWP1 cells were treated with 0.5 μg·mL^−1^ doxy for 48 h before harvesting. GAPDH was used as loading control. PARP cleavage was measured to evaluate cell viability. Three independent experiments were carried out. (D) Detection of ROS (reactive oxygen species) by the H_2_DCFDA assay in control and WWP1‐depleted NB4 cells in the absence or in the presence of 10 mm NAC. NB4/Tet‐On/shWWP1 cells were treated with 0.5 μg·mL^−1^ doxy for 48 h before incubation with 1 μm H_2_DCFDA. ROS levels are expressed as fold increase in fluorescence intensity. Values represent the mean ± SD from three independent experiments. (E–G) Evaluation of cell death in control and WWP1‐depleted NB4 cells. NB4/Tet‐On/shWWP1 cells were treated with 0.5 μg·mL^−1^ doxy for 72 h. (E) Percentages of double DAPI/annexin‐V negative (live) and DAPI negative/annexin‐V positive (apoptotic) cells were assessed by flow cytometry after staining with FITC annexin‐V conjugates and DAPI. Values represent the mean ± SD from three independent experiments. (F) Percentage of apoptotic cells in the sub G1 phase was evaluated by flow cytometry after propidium iodide staining. Values represent the mean ± SD from three independent experiments (G) Representative western blot analysis of cleaved PARP1 and caspase‐3 proteins. GAPDH was used as loading control. Three independent experiments were carried out. Statistically significant differences were calculated by Student's *t*‐test. *P < 0.05, **P < 0.01, ***P < 0.001.

We next used quantification of DNA breaks by means of comet assay as an indirect parameter of oxidative stress. The comet assay revealed accumulation of DNA strand breaks in shWWP1 relatively to control cells (Fig. 2A,B). This finding was corroborated by the enhanced expression of phosphorylated histone H2AX (γ‐H2AX), a sensitive molecular marker of DNA damage (Fig. 2C). Ataxia telangiectasia mutated protein (ATM) was also significantly activated by WWP1 silencing along with BRCA1 accumulation (Fig. 2C), suggesting an attempt of the cells to activate mechanisms of DNA repair. To assess whether the increase in ROS levels may be responsible for driving DNA damage accumulation, we used the antioxidant *N*‐acetyl cysteine (NAC) as a ROS scavenger (Fig. 2D). Notably, WWP1‐dependent accumulation of DNA damage was reversed by treatment with NAC (Fig. 2A,B), confirming that WWP1 depletion induces DNA damage through ROS generation. Coherently with increased levels of endogenous DNA damage, cell viability starts decreasing at later time points (72 h) after WWP1 inactivation (Fig. 2E–G and Fig. S1E–H).

### 3.3 TXNIP is a substrate for the ubiquitination activity of WWP1

**Fig. 3:**
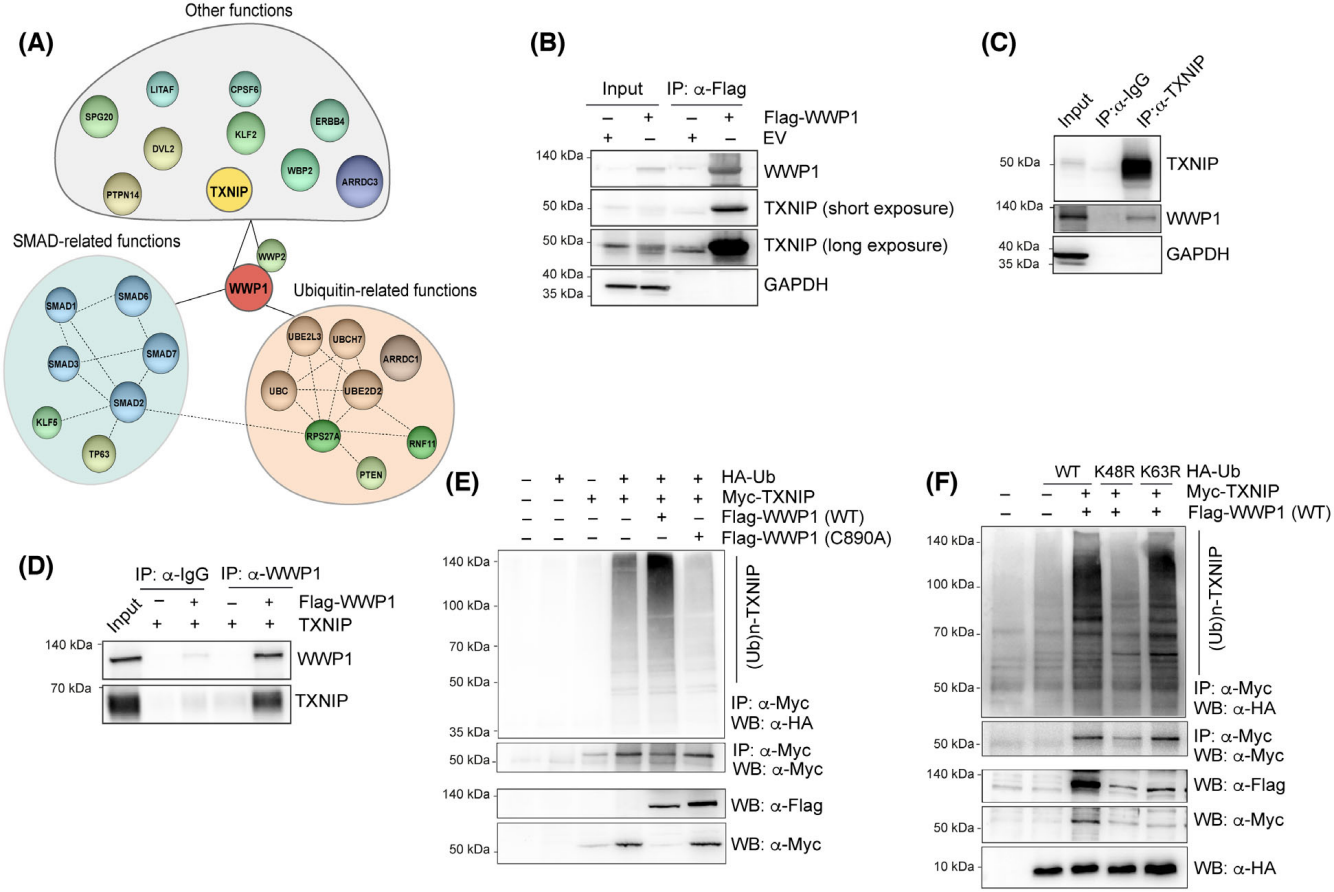
WWP1 directly binds TXNIP and promotes its ubiquitination. (A) Graphical representation of the protein–protein interaction network for the human WWP1 protein obtained using the online database STRING. (B) Representative western blot for the co‐immunoprecipitation assays. HEK293T cells were transfected with Flag‐WWP1 or control empty vector (EV). Cellular extracts were pulled down with anti‐Flag antibody and then analyzed by western blot with anti‐TXNIP and anti‐Flag antibodies. Three independent experiments were carried out. (C) Representative western blot showing binding of endogenous TXNIP and WWP1 in NB4 cells. Cellular lysates were immunoprecipitated with anti‐TXNIP or with an IgG isotype control antibody, and then subjected to western blot with anti‐WWP1 and anti‐TXNIP antibodies. GAPDH was used as loading control. Three independent experiments were carried out. (D) *In vitro* pull‐down of WWP1 to confirm direct interaction between WWP1 and TXNIP. Recombinant Flag‐WWP1 (300 ng) and His‐TXNIP (300 ng) proteins were incubated overnight. WWP1 was then immunoprecipitated by using anti‐WWP1 antibody and the immunocomplexes were subjected to western blot with anti‐TXNIP antibody. Isotypic IgG were used as a negative control. Three independent experiments were carried out. (E) Representative western blot analysis of the *in vivo* ubiquitination of TXNIP by WWP1. HEK293T cells were co‐transfected with wild‐type Flag‐WWP1 (WT), the ligase‐dead Flag‐WWP1 mutant (C890A) or control vector, along with Myc‐TXNIP and HA‐ubiquitin (HA‐Ub). Ubiquitinated TXNIP was assessed by immunoprecipitation with anti‐Myc antibody, followed by detection of ubiquitinated species using anti‐HA antibody. Three independent experiments were carried out. (F) Effect of the K48R and K63R ubiquitin mutants on WWP1‐mediated TXNIP polyubiquitination. HEK293T cells were co‐transfected with wild‐type Flag‐WWP1 (WT), TXNIP and either wild‐type HA‐Ub or the indicated ubiquitin mutants. Cellular extracts were subjected to *in vivo* ubiquitination assay (as above). Three independent experiments were carried out.

Oxidative stress may occur either by increased ROS generation or by decreased antioxidant defense mechanisms, including alterations in GSH content and in the Trx system. Because we did not observe changes in the levels of GSH in control versus WWP1‐depleted cells (Fig. 1A), we sought to investigate a potential link between WWP1 and the Trx system. Hence, we next exploited the STRING database to search for potential thioredoxin system‐related candidates. Protein interaction analysis revealed that WWP1 potentially interacts with the Trx inhibitor, TXNIP (Fig. 3A and Table S1). In addition, TXNIP contains two proline‐rich domain (PPxY) motifs (Fig. S2A) that generally mediate substrate recruitment by the HECT E3s. We therefore hypothesized that WWP1 maintains cellular ROS homeostasis through TXNIP regulation. To investigate the interaction of WWP1 and TXNIP, we carried out immunoprecipitation assays in HEK293T cells transiently transfected with Flag‐WWP1. When WWP1 was precipitated with anti‐FLAG antibody, endogenous TXNIP was detected in the immune complexes (Fig. 3B). The interaction between WWP1 and TXNIP was also confirmed at endogenous level in leukemic cells (Fig. 3C and Fig. S2B). To examine whether TXNIP directly interacts with WWP1, we performed an *in vitro* pulldown assay using recombinant proteins and found that TXNIP was able to strongly bind WWP1 (Fig. 3D). *In vivo* ubiquitination assays showed that wild‐type WWP1 (WT) is capable to induce TXNIP poly‐ubiquitination (Fig. 3E). On the contrary, the expression of an enzymatically inactive WWP1 mutant (C890A), in which the catalytic cysteine has been converted to alanine (Cys890→Ala), abrogated this effect (Fig. 3E), indicating that the catalytic activity of WWP1 is indispensable for TXNIP ubiquitination. Proteasomal degradation of TXNIP has been shown to be influenced by the extracellular signal‐regulated protein kinase (ERK) through a not yet identified E3 [44]. ERK promotes phosphorylation of TXNIP at Thr349 residue within in the PXTP motif located within its C‐terminal domain. These findings suggest a potential requirement of ERK phosphorylating activity for WWP1‐mediated ubiquitination of TXNIP. However, ERK inhibition did not affect the ability of WWP1 to ubiquitinate TXNIP and to negatively regulate its protein stability (Fig. S2C), implicating alternative molecular mechanisms by which TXNIP phosphorylation drives its ubiquitination and proteasomal degradation.

To determine the type of ubiquitin linkage implicated in TXNIP modification by WWP1, we evaluated the ability of ubiquitin mutants containing a single lysine to arginine mutation at positions 48 (K48R) and 63 (K63R) to ubiquitinate TXNIP. These ubiquitin mutants are expected to abolish K48 and K63 ubiquitin linkage formation on protein substrates, respectively. As shown in Fig. 3F, WWP1‐mediated TXNIP ubiquitination was prevented in the presence of the K48R ubiquitin mutant, whereas it was unaffected in K63R ubiquitin mutant expressing cells, implicating K48 in polyubiquitin chain formation by WWP1. Furthermore, using an *in vitro* ubiquitination assay, we found that WWP1 was unable to affect TXNIP ubiquitination (Fig. S2D), suggesting that TXNIP modification by WWP1 requires additional cellular components such as adaptors or other E3s.

### 3.4 WWP1 silencing leads to TXNIP stabilization and functional activation

**Fig. 4:**
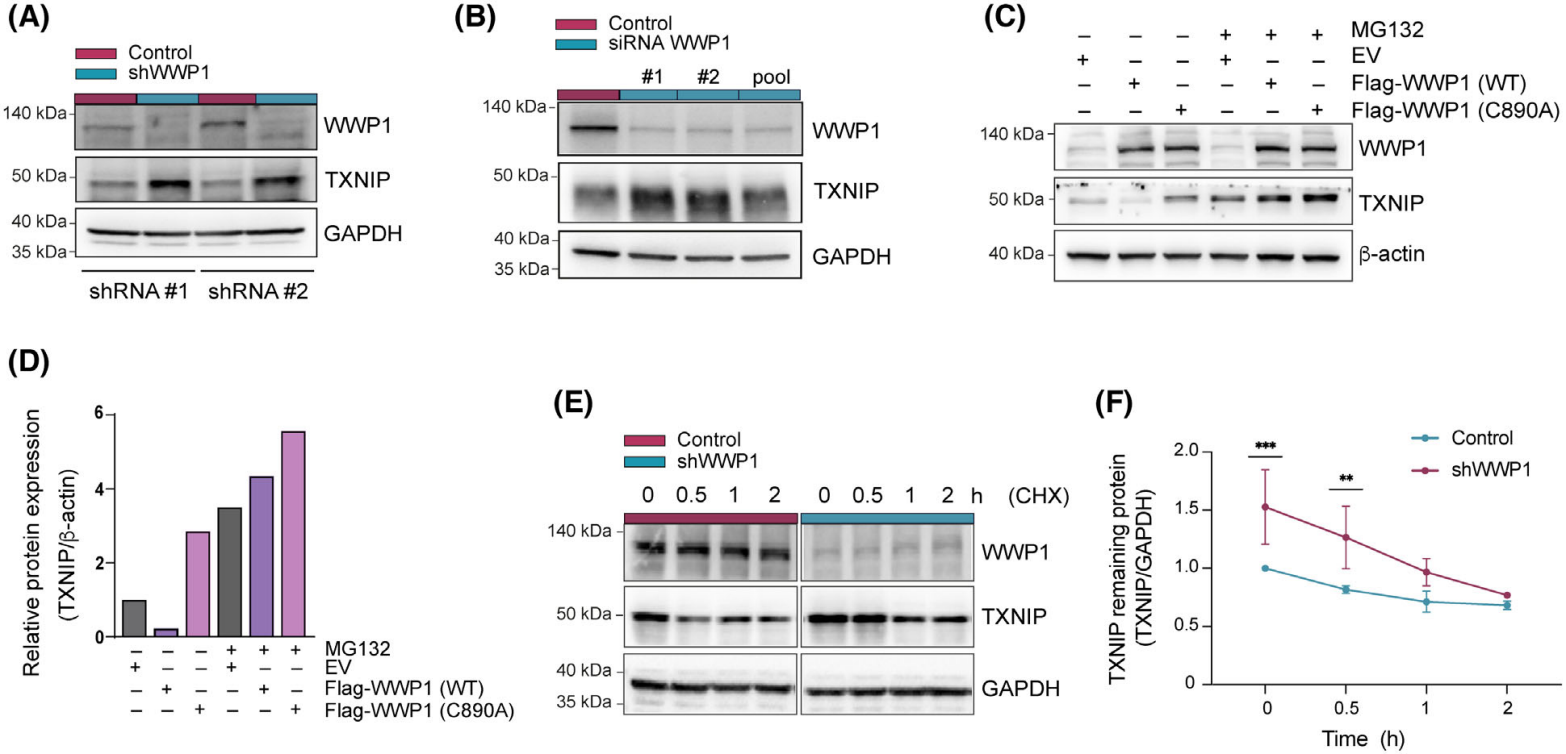
WWP1 Induces ubiquitin‐proteasome‐dependent degradation of TXNIP. (A, B) WWP1 knockdown increases TXNIP protein levels in AML (acute myeloid leukemia) blasts. (A) Representative western blot analysis of TXNIP protein levels in control and WWP1‐depleted cells. To achieve WWP1 inactivation, an additional NB4/Tet‐On/shWWP1 cell clone was used. Both clones were treated with 0.5 μg·mL^−1^ doxy for 48 h before harvesting. GAPDH was used as loading control. Three independent experiments were carried out. (B) Representative western blot analysis of TXNIP protein levels following siRNA‐mediated knockdown of WWP1 in OCI‐AML3 cells. Cells were transfected with a non‐targeting control siRNA or with one of two individual siRNAs (siWWP1 #1 or siWWP1 #2), or with a pool of siRNAs, siWWP1 #1 and siWWP1 #2, for 48 h. Three independent experiments were carried out. (C, D) The decrease in TXNIP protein levels upon WWP1 overexpression is dependent on its E3 ligase activity. Representative western blot analysis in HEK293T cells of Flag‐WWP1 and TXNIP protein levels (C) and related quantification of TXNIP amounts (D). HEK293T cells were co‐transfected with either wild‐type Flag‐WWP1(WT) or the C890A mutant, or control empty vector (EV). Four hours before harvesting, cells were either left untreated or incubated with 10 μm MG132. Forty‐eight hours post‐transfection, cells were harvested, lysed, and equal amounts of protein extracts were subjected to western blot analysis with the indicated antibodies. β‐Actin was used as loading control. Three independent experiments were carried out. (E, F) Analysis of TXNIP degradation by CHX chase. Three independent experiments were carried out. (E) Representative western blotting analysis of WWP1 and TXNIP protein levels in control and shWWP1 cells before and 0.5, 1 and 2 h after protein synthesis blockade by the addition of CHX. GAPDH was used as loading control. (F) Relative changes in TXNIP half‐life were quantified as mean ± SD from three independent experiments using imagej software and normalized to GAPDH. Values were normalized to the time 0 of control cells as 1. Statistical significance was calculated using one‐way ANOVA test. **P < 0.01, ***P < 0.001.

We next evaluated the outcome of TXNIP modification by WWP1. We found increased abundance of TXNIP following WWP1 inactivation in AML cells (Fig. 4A,B), suggesting a potential role of this enzyme in the alteration of the redox state induced by WWP1 silencing. Coherently, overexpression of WT WWP1 promoted TXNIP down‐regulation in HEK293T (Fig. 4C,D). On the contrary, ectopic expression of the WWP1‐C890A mutant was unable to modulate TXNIP protein levels (Fig. 4C,D), further supporting the evidence that the enzymatic activity of WWP1 is indispensable for TXNIP regulation. Pre‐treatment of WWP1‐overxpressing cells with the proteasomal inhibitor MG132 restored TXNIP protein levels indicating that its downregulation occurs via proteasome‐dependent degradation. We next sought to measure the rate of TXNIP degradation through the assessment of its steady‐state turnover in control and WWP1‐depleted cells after translation inhibition with cycloheximide (CHX). Inactivation of WWP1 significantly prolonged the half‐life of TXNIP protein compared with control cells (Fig. 4E,F).

**Fig. 5:**
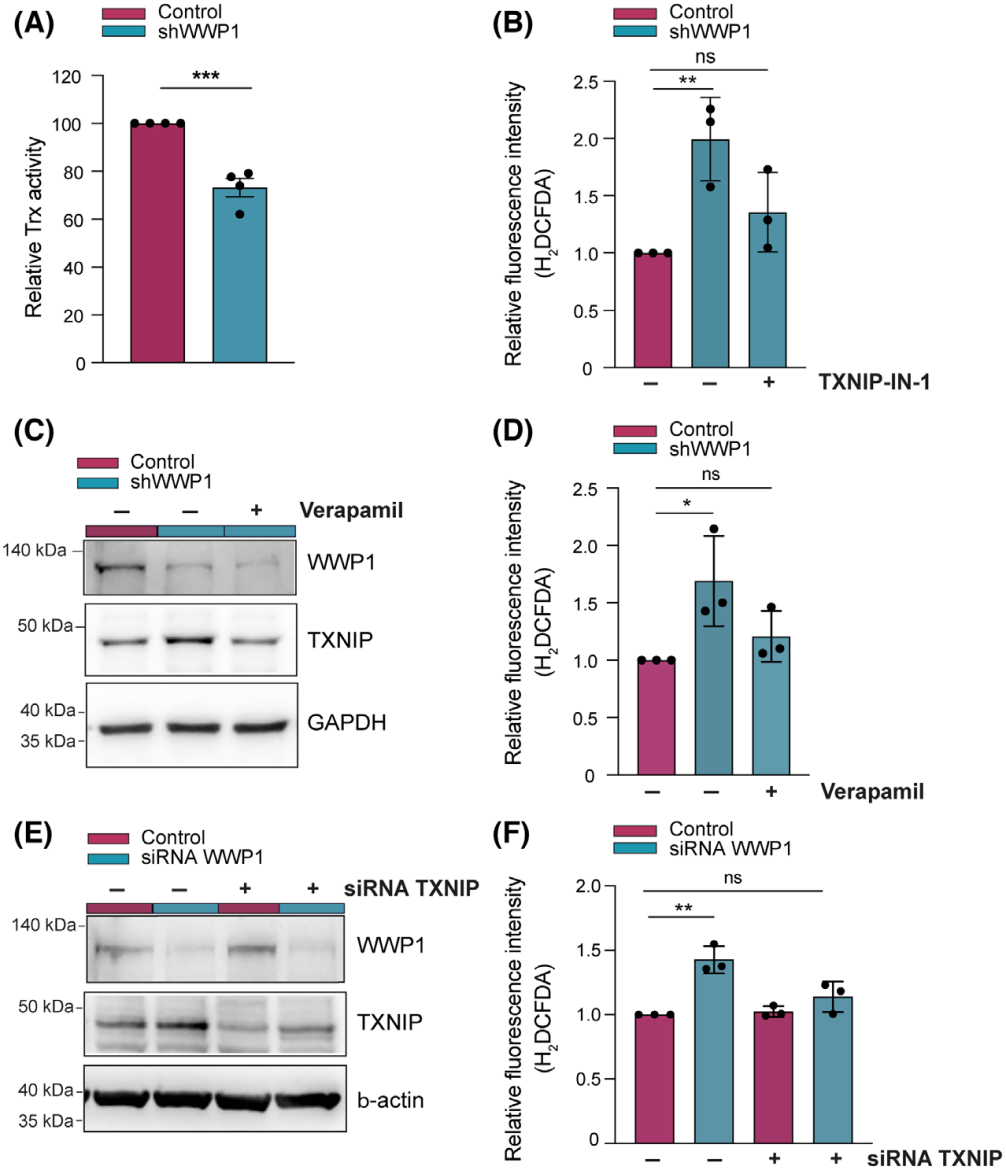
WWP1 Silencing potentiates TXNIP pro‐oxidative functions. (A) Trx activity was measured in control and WWP1‐depleted NB4 cells at 412 nm. NB4/Tet‐On/shWWP1cells were treated with 0.5 μg·mL^−1^ doxy for 48 h before harvesting. Four separate measurements were performed and indicated as mean ± SD. (B) Measurement of ROS (reactive oxygen species) levels in control and WWP1‐depleted NB4 cells by the H2DCFDA assay. NB4/Tet‐On/shWWP1 cells were left untreated or treated with 0.5 μg·mL^−1^ doxy for 48 h in the absence or in the presence of 5 μm TXNIP‐IN‐1 inhibitor for 24 h. ROS levels are expressed as fold increase in fluorescence intensity. Values represent the mean ± SD from three independent experiments. (C) Representative western blot analysis of TXNIP protein levels in response to verapamil. To achieve WWP1 inactivation, NB4/Tet‐On/shWWP1 cells were treated with 0.5 μg·mL^−1^ doxy for 48 h and either left untreated or incubated with 50 μm verapamil for 16 h before harvesting. GAPDH was used as loading control. Three independent experiments were carried out. (D) Intracellular ROS detection in control and WWP1‐depleted NB4 cells upon verapamil treatment (see above). ROS were measured by the H_2_DCFDA assay and are expressed as fold increase in fluorescence intensity. Values represent the mean ± SD from three independent experiments. (E) Representative Western blot analysis of TXNIP protein levels following siRNA‐directed gene silencing of WWP1 and TXNIP in OCI‐AML3 cells. Cells were transfected with a non‐targeting control siRNA or with a pool of two individual siRNAs (siTXNIP #1 and siTXNIP #2) in combination or not with a pool of two individual siRNAs (siWWP1 #1 and siWWP1 #2) for 48 h. β‐Actin was used as loading control. Three independent experiments were carried out. (F) Effect of TXNIP siRNA delivery on ROS production triggered by WWP1 silencing in OCI‐AML3 cells. Cells were transfected with a non‐targeting control siRNA or with a pool of two individual siRNAs (siTXNIP #1 and siTXNIP #2) in combination or not with a pool of two individual siRNAs (siWWP1 #1 and siWWP1 #2) for 48 h. ROS were measured by the H_2_DCFDA assay and are expressed as fold increase in fluorescence intensity. Values represent the mean ± SD from three independent experiments. Statistical significance was analyzed by Student's *t*‐test. *P < 0.05, **P < 0.01, ***P < 0.001.

Based on these data, we hypothesized that WWP1 maintains cellular ROS homeostasis through TXNIP degradation, which ultimately leads to increased Trx enzymatic activity. Hence, we next performed an insulin disulfide reductase assay to elucidate the impact of WWP1 on the Trx system. Figure 5A shows that Trx enzymatic activity decreases in AML cells upon silencing of WWP1. Altogether, these findings indicate that WWP1 restrains intracellular ROS generation through TXNIP proteasomal degradation and subsequent activation of antioxidative function of Trx. To further validate these findings, we assessed the effect of TXNIP inhibition in WWP1‐depleted AML cells. For this purpose, we undertook three different experimental approaches. We exploited the TXNIP‐IN‐1 compound, a TXNIP‐Trx complex inhibitor [45] and found that it could rescue the increase of cellular ROS elicited by WWP1 inactivation in NB4 cells (Fig. 5B). We also treated NB4 cells with Verapamil, a calcium channel blocker drug that has been reported to suppress the transcription of TXNIP mRNA [46, 47]. The exposure of WWP1‐depleted NB4 cells to Verapamil restored the levels of TXNIP to those detected in control cells (Fig. 5C) and reduced the levels of ROS resulting from WWP1 silencing (Fig. 5D). Furthermore, gene silencing of TXNIP in OCI‐AML3 cells alleviated ROS production triggered by concomitant inactivation of WWP1 expression (Fig. 5E,F), proving a necessary role of TXNIP in mediating the prooxidant activity of WWP1 silencing in AML cells.

**Fig. 6:**
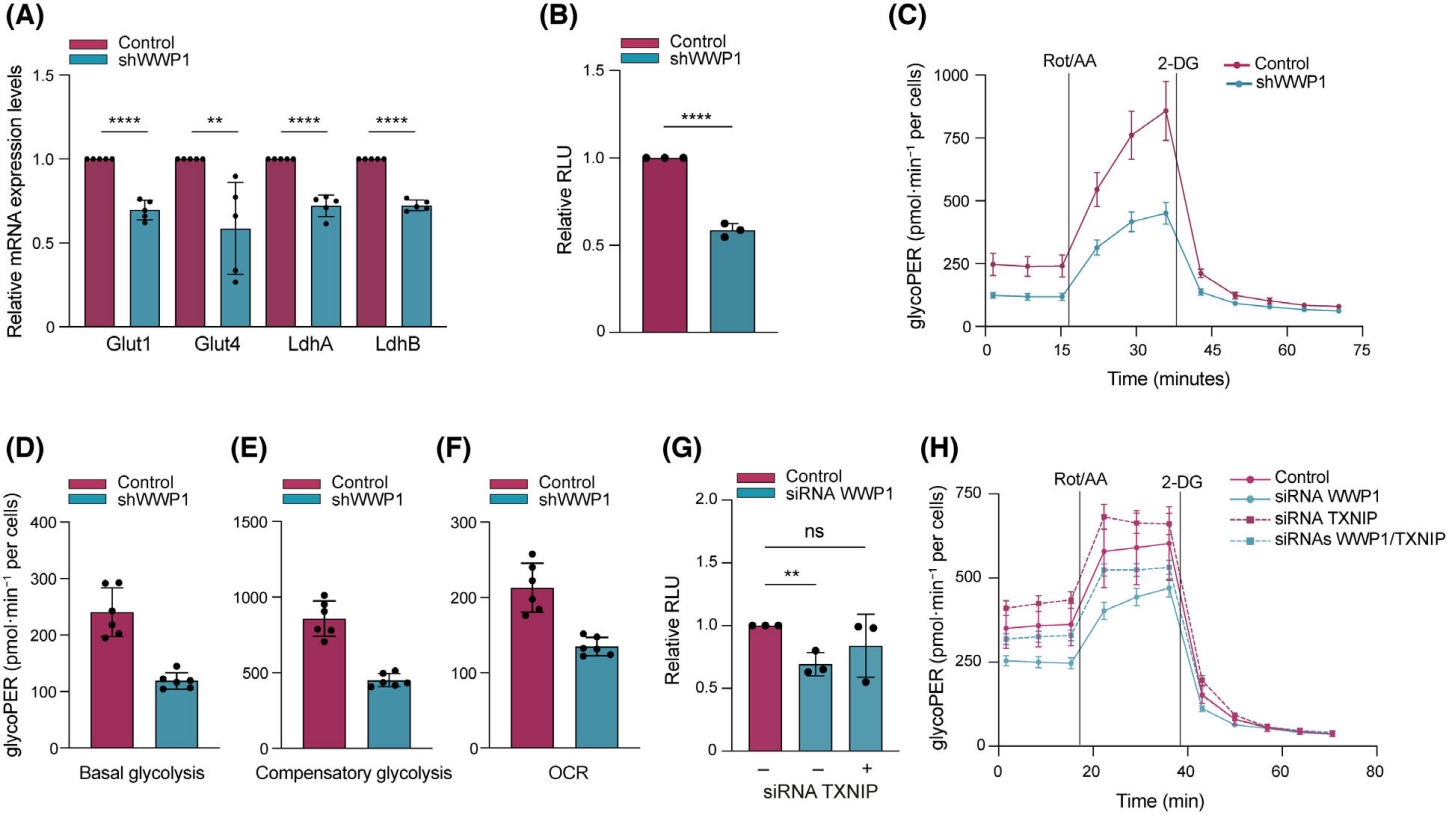
WWP1 affect the ability of TXNIP to modulate glucose metabolism. (A) The mRNA levels of GLUT1, GLUT4, LDHA and LDHB, in control and shWWP1 NB4 cells were analyzed by RT‐qPCR. The relative abundances of transcripts were quantified and normalized to TBP (Tata‐Box Binding Protein). Values represent the mean ± SD from five independent experiments. (B) Glucose uptake is reduced in WWP1‐depleted cells. Cells were treated with 0.5 μg·mL^−1^ doxy for 48 h to induce shWWP1 expression. Intracellular glucose transport was measured with a bioluminescent glucose uptake assay and reported as luminescence intensity in RLU (Relative light unit). (C–E) Glycolytic function measured by Seahorse analysis as glycolytic proton efflux rate (glycoPER) in control and WWP1‐depleted NB4 cells. Cells were treated with 0.5 μg·mL^−1^ doxy for 48 h to induce shWWP1 expression. Three independent experiments were carried out. (C) Representative glycoPER curve (pmol·min^−1^ per cells) obtained after injections of Rot/AA (Rotenone/antimycin A) (0.5 μm), and 2‐DG (2‐deoxyglucose) (50 mm). Values represent the mean ± SD from six technical replicates. Representative basal (D) and (E) compensatory glycolytic PER (pmol·min^−1^ per cells) presented as bar graphs showing the mean ± SD from six technical replicates. (F) Effect of WWP1 inactivation on oxygen consumption rates (OCR) in control and WWP1‐depleted NB4 cells. Values represent the mean ± SD from six technical replicates. Three independent experiments were carried out. (G, H) Evaluation of the combination effect of WWP1 and TXNIP siRNAs on glucose up‐take and consumption. Cells were transfected with siWWP1 #1 and siWWP1 #2, or with siTXNIP #1 and siTXNIP #2, or with the combination of the 4 siRNAs for 48 h. (G) Intracellular glucose transport was measured with a bioluminescent glucose uptake assay. Values represent the mean ± SD from three independent experiments. (H) Glycolytic function was measured by Seahorse analysis and represented as glycoPER curve (pmol·min^−1^ per cells) after injections of Rot/AA (0.5 μm), and 2‐DG (50 mm). Three independent experiments were carried out. Values represent the mean ± SD from six technical replicates of a representative experiment. Statistical significance was analyzed by Student's *t*‐test. **P < 0.01, ****P < 0.0001.

To further assess the ability of WWP1 to regulate TXNIP biological functions, we evaluated the expression of enzymes related to glucose uptake and metabolism, such as the glucose transporters GLUT1 and GLUT4, hexokinase 2 (HK2), lactate dehydrogenase A (LDHA), and B (LDHB), that are known to be transcriptionally regulated by TXNIP [27]. Except for HK2 (data not shown), the mRNA levels of these enzymes decreased in WWP1‐depleted cells (Fig. 6A). In line with these data, we found decreased glucose uptake upon WWP1 downregulation (Fig. 6B).

To further dissect the contribution of WWP1 to TXNIP regulation, we evaluated the glycolytic rates of control and WWP1‐depletd cells analyzing their glycolytic proton efflux rate (glycoPER, also referred as basal glycolysis), that corresponds to glycolytic‐produced lactate‐derived acidification. WWP1 downregulation significantly reduced basal and compensatory glycolysis of NB4 cells (Fig. 6C–E and Fig. S3A–C). Coherently, we found that ablation of WWP1 leads to decreased oxidative phosphorylation measured as oxygen consumption rate (OCR) (Fig. 6F and Fig. S3D) and reduced glycolytic and mitochondrial ATP production rates (Fig. S3E). Overall, these findings indicate that WWP1 depletion impairs glucose uptake and consumption via TXNIP regulation. To formally prove the relevance of the WWP1/TXNIP functional axis in glucose metabolism regulation, we measured glucose uptake and glycolytic rates following TXNIP silencing in WWP1‐depleted AML cells. As shown in Fig. 6, combinatorial WWP1 and TXNIP siRNA delivery to AML cells rescued the siRNA WWP1‐induced defects in glucose uptake and glycolysis (Fig. 6G,H and Fig. S3F,G).

TXNIP regulates glucose metabolism through either HIF1α‐ independent [30, 48] or dependent mechanisms [49]. The latter are achieved via destabilization of HIF1α by TXNIP in both hypoxia and normoxia conditions [49]. To investigate whether the alterations detected in the absence of WWP1 in the transcription of the glycolytic genes were HIF1α‐dependent [50], we measured HIF1α protein levels in control and WWP1‐depeted cells under normoxic conditions. We did not observe differences in HIF1α protein amounts in shWWP1 relatively to control NB4 cells (Fig. S3H), implying that WWP1 influences the regulatory activity of TXNIP on glucose metabolism independently of HIF1α.

## 4 Discussion

Here, we demonstrate that TXNIP stability is negatively regulated by WWP1 in AML cells, a tumor in which additional therapeutic targets are needed [51, 52]. TXNIP is directly recruited by WWP1 and acts as a substrate for its ubiquitination activity. Polyubiquitination of TXNIP by WWP1 ultimately results in its proteasomal degradation. The HECT‐type E3 ITCH was previously reported to target TXNIP for ubiquitin‐dependent proteasomal degradation [53]. Ohtake et al. [54] reported that ITCH‐dependent K63 ubiquitination of TXNIP functions as an anchor for subsequent seeding of K48/K63 branched chains. This mechanism requires the recruitment by ITCH of an additional HECT‐type E3, UBR5 that is responsible for further elongation of K48/K63 branched ubiquitin on TXNIP. On the contrary, we found that the K48R, but not the K63R ubiquitin mutant, failed in promoting TXNIP ubiquitination by WWP1, demonstrating that K48, and possibly additional ubiquitin linkages, might be responsible for TXNIP modification. In addition, we could not detect TXNIP ubiquitination in reconstituted *in vitro* assays, suggesting that, similarly to ITCH, WWP1 may require adaptors, modifications and/or additional E3s, such as UBR5, to modify TXNIP. In our study, we could rule out a contribution of ERK‐mediated phosphorylation to the regulation of TXNIP ubiquitination and stability by WWP1. Nevertheless, the precise biochemical mode of polyubiquitination of TXNIP by WWP1 remains to be fully elucidated.

Accumulation of TXNIP in WWP1‐depleted cells leads to a decrease in Trx oxidoreductase activity, consequent enhancement of both cellular mitochondrial ROS content. Unbalanced redox homeostasis is eventually responsible for DNA damage accumulation and induction of cell death of AML cells. In parallel, TXNIP stabilization upon WWP1 depletion hampers glucose up‐take and glucose metabolism of leukemia cells, with subsequent reduction of ATP production.

**Fig. 7:**
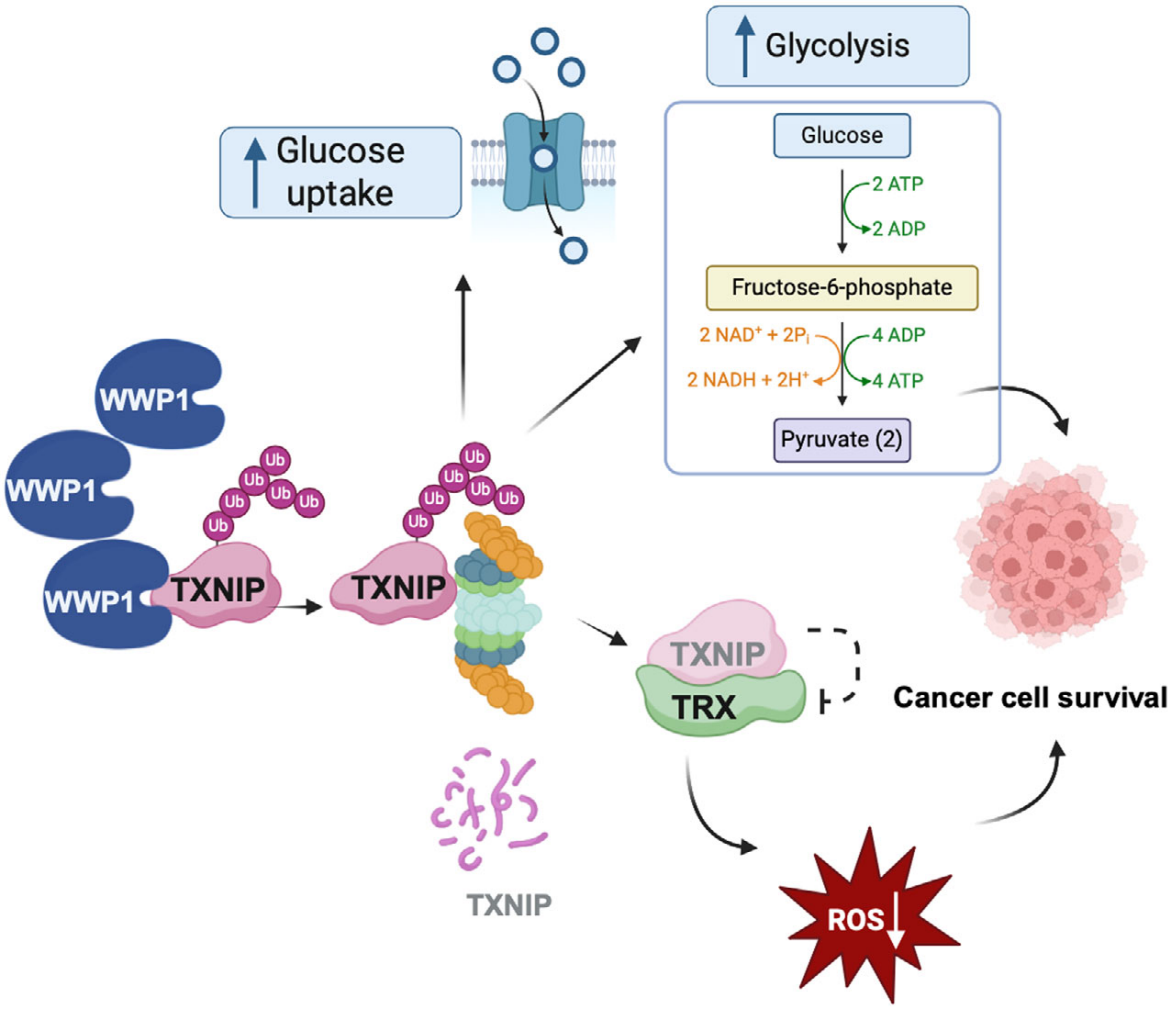
Schematic model depicting the potential outcome of WWP1 overexpression in AML patients. The higher availability of WWP1 in AML (acute myeloid leukemia) blasts would accelerate TXNIP proteasomal removal thus hampering the inhibition exerted on Trx. Enhanced activity of the Trx antioxidant system would then lower the amount of cellular ROS (reactive oxygen species) thus sustaining cancer cell fitness. Concomitantly, sustained degradation of TXNIP would restricts its ability to inhibit intracellular glucose transport and consumption, hence contributing to promote cancer cell survival and proliferation.

According to our findings, we can propose a model according to which the overexpression of *WWP1* in AML blasts could lead to increased Trx activity through accelerated TXNIP degradation, thus lowering intracellular ROS levels and favoring survival of cancer cells (Fig. 7). In line with the ability of TXNIP to act as a potent negative regulator of glucose uptake, glycolytic gene expression and aerobic glycolysis, we found that TXNIP accumulation upon WWP1 inactivation was capable of inhibiting glucose up‐take and glycolysis. All together, these findings suggest that TXNIP reduced availability, as a result of WWP1 deregulation, would promote proliferation and survival of AML blasts by buffering cellular ROS levels and fostering glucose uptake and consumption (Fig. 7).

Redox homeostasis plays a key role in the response of cancer cells to chemotherapeutic drugs. We can also speculate that under conditions of acute oxidative stress, such as following exposure to chemotherapy, since *WWP1*‐overexpressing AML cells should possess higher Trx oxidoreductase activity, they would be more proficient in ROS scavenging. This alteration in the redox homeostasis would then limit the cytotoxicity of ROS‐inducing chemotherapeutic agents and as such the response of tumor cells. Coherently, *TXNIP* overexpression has been found to decrease chemoresistance by increasing drug‐induced ROS and DNA damage levels in tumor cells [26, 55]. Among the other tumors, TXNIP expression is down‐regulated in chronic myeloid leukemia (CML) in response to activated BCR‐ABL fusion protein that stimulates glucose metabolism, mainly by increasing glucose transporter exposure at the plasma membrane. On the contrary, the BCR‐ABL inhibitor imatinib acts by decreasing the surface localization of glucose transporters. In CML, TXNIP is necessary for the imatinib inhibitory effect on leukemia cell growth [34]. Indeed, restoration of *TXNIP* expression sensitizes CML blasts to imatinib exposure, possibly via potentiation of glucose metabolism blockade [34].

## 5 Conclusions

Here, we demonstrate that the WWP1/TXNIP functional axis is crucial for the regulation of the cellular redox state and glucose metabolism of AML cells. Both redox and metabolic imbalances may contribute to decrease cell viability of cancer cells and to their response to chemotherapy. Future studies are needed to assess the involvement of the WWP1/TXNIP crosstalk in the regulation of leukemic cell sensitivity to anti‐cancer therapies.

## Conflict of interest

The authors declare no conflict of interest.

## Author contributions

FB conceived the project, designed experiments, analyzed data, and wrote the paper. SG, YL, RP, CF, FC, ADA, AS, VM and Ji Z performed experiments, acquired, and analyzed data. MA, SR, AMT, JZ, YS, EC and GM analyzed data and interpreted the results. All the Authors revised the manuscript and approved the final version.

### Peer review

The peer review history for this article is available at https://www.webofscience.com/api/gateway/wos/peer‐review/10.1002/1878‐0261.13722.

## Supporting information


**Fig. S1.** Oxidative stress induced by WWP1 inactivation triggers apoptotic cell death.
**Fig. S2.** WWP1 binds TXNIP and promotes its ubiquitination independently of ERK‐mediated phosphorylation.
**Fig. S3.** WWP1 influences TXNIP‐mediated regulation of glucose uptake and consumption.

**Table S1.** List of the WWP1 interactors.

**Table S2.** List of primers used in the study.

## Data Availability

Data that support the findings of this study are available from the corresponding author [bernasso@uniroma2.it] upon reasonable request.
