# Supplementary material for: Thioredoxin‐interacting protein (TXNIP) is a substrate of the NEDD4‐like E3 ubiquitin‐protein ligase WWP1 in cellular redox state regulation of acute myeloid leukemia cells
Source: Mol Oncol. 2024 Oct 4;19(1):133–50. doi: 10.1002/1878-0261.13722 (PMC11705725; doi:10.1002/1878-0261.13722)
Supplement: Supplementary file 1 — Fig. S1. Oxidative stress induced by WWP1 inactivation triggers apoptotic cell death. Fig. S2. WWP1 binds TXNIP and promotes its ubiquitination independently of ERK‐mediated phosphorylation. Fig. S3. WWP1 influences TXNIP‐mediated regulation of glucose uptake and consumption. [file MOL2-19-133-s003.zip › supplementary figure legends.docx]

**Figure S1**. **Oxidative stress induced by WWP1 inactivation triggers apoptotic cell death.** (A) Representative FACS analysis of ROS production in control and WWP1-depleted NB4 cells by the H_2_DCFDA assay. NB4/Tet-On/shWWP1 cells were treated with 0,5 μg/mL doxy for 48 hours before incubation with 1 μM H_2_DCFDA. (B) Detection of ROS in control and WWP1-depleted OCI-AML3 by the H_2_DCFDA assay. OCI-AML3 cells were either transfected with a non-targeting control siRNA or with a pool of two individual siRNAs against WWP1 for 48 hours before 20 minutes incubation with 1 μM H_2_DCFDA. ROS levels are expressed as fold increase in fluorescence intensity. Values represent the mean ± SD from 3 independent experiments. The results are expressed as the mean fluorescence of 3 independent experiments ± SD. (C) Mitochondrial membrane potential changes in WWP1 depleted OCI-AML3 cells 48 hours after transfection using flow cytometer after JC-1 staining. Cells were transfected with a non-targeting control siRNA or with a pool of two individual siRNAs: siWWP1 #1 and siWWP1 #2. Values represent the mean ± SD from 4 independent experiments. (D) CellTiter-Glo® luminescent cell viability assay in NB4 cells following WWP1 inactivation. Luminescence measurements are expressed as RLU (Relative Light Unit). Values represent the mean ± SD from three independent experiments. (E-H) Induction of apoptotic cell death in WWP1-depleted leukemic cells. Representative annexin V/DAPI staining profile (E) and flow cytometric cell cycle analysis (F) of control and WWP1-depleted NB4 cells. (G) Percentages of double DAPI/annexin-V negative (live) and DAPI negative/annexin-V positive (apoptotic) OCI-AML3 cells following 48 hours transfection with WWP1 siRNAs. Apoptosis was assessed by flow cytometry after staining with FITC annexin-V conjugates and propidium iodide. Values represent the mean ± SD from 4 independent experiments. (H) Representative Western blot analysis of cleaved PARP1 and caspase-3 proteins measured 72 hours following delivery of WWP1 siRNA in OCI-AML3 cells (see above). GAPDH was used as loading control. Three independent experiments were carried out. *p < 0.05, ***p < 0.001.

**Figure S2.** **WWP1 binds TXNIP and promotes its ubiquitination independently of ERK-mediated phosphorylation.** (A) Primary sequence of *Homo sapiens* TXNIP (isoform_1) in which the two conserved PPXY motifs are highlighted in yellow. (B) Representative Western blot showing binding of endogenous TXNIP and WWP1 in OCI-AML3 cells. Cellular lysates were immunoprecipitated with anti-TXNIP or with an IgG isotype control antibody, and then subjected to Western blot with anti-WWP1 and anti-TXNIP antibodies. GAPDH was used as loading control. Three independent experiments were carried out. (C) Effect of ERK inhibition on WWP1-mediated regulation of TXNIP protein stability and ubiquitination. Representative Western blot analysis of *in vivo* ubiquitination of TXNIP by WWP1. HEK293T cells were co-transfected with Flag-WWP1 or control vector, along with Myc-TXNIP and HA-ubiquitin (HA-Ub). Treatment with SCH772984 was carried out at the concentration of 250 nM for 24 hours. Ubiquitinated TXNIP was assessed by immunoprecipitation with anti-Myc antibody, followed by detection of ubiquitinated species using anti-HA antibody. Three independent experiments were carried out. (D) WWP1 is unable to directly ubiquitinate TXNIP *in vitro*. Recombinant TXNIP was incubated with WWP1 in the presence of E1, E2 (UbcH7), and ubiquitin. Following the ubiquitination reaction (which doesn’t take place at T0°C, lane 1, but at T30°C, lanes 2 and 3), the TXNIP-ubiquitin conjugates were detected by Western blot with anti-TXNIP antibody. The right panel shows WWP1 auto-ubiquitination under the same experimental condition.

**Figure S3.** **WWP1 influences TXNIP-mediated regulation of glucose uptake and consumption.** (A-D) Glycolytic function measured by Seahorse analysis as glycoPER in control and WWP1-depleted OCI-AML3 cells. Cells were transfected with siRNAs for 48 hours to reduce WWP1 expression. (A) Representative glycoPER curve (pmol/min/cells) obtained after injections of Rot/AA (0.5 μM), and 2-DG (50 mM). Representative basal (B) and (C) compensatory glycoPER (pmol/min/cells) presented as bar graphs. (D) Effect of WWP1 inactivation on OCR in control and WWP1-depleted OCI-AML3 cells. Values represent the mean ± SD from eight technical replicates. Three independent experiments were carried out. (E) ATP production rate measured in control and WWP1-depleted NB4 cells by Seahorse analysis and represented as glycolytic or mitochondrial ATP production rate (pmol/min/cells). (F, G) Evaluation of the combination effect of WWP1 and TXNIP siRNAs on glucose consumption. Cells were transfected with a non-targeting control siRNA, or with a pool of siWWP1 #1 and siWWP1 #2, or with a pool of siTXNIP #1 and siTXNIP #2, or with a combination of both siWWP1 and siTXNIP siRNA pools. Glycolytic function was measured by Seahorse analysis and represented as basal (F) and compensatory (G) glycolysis (pmol/min/cells). Values represent the mean ± SD from 5 technical replicates. Three independent experiments were carried out. (H) Western blot analysis of HIF-1α protein levels in control and WWP1-depleted cells. NB4/Tet-On/shWWP1 cells were treated with 0,5 μg/mL doxy for 48 hours before harvesting. GAPDH was used as loading control. CoCl2 treatment of NB4/Tet-On/shWWP1 cells was used as positive control for HIF-1α induction.
