# Supplementary figures and images for: Thioredoxin‐interacting protein (TXNIP) is a substrate of the NEDD4‐like E3 ubiquitin‐protein ligase WWP1 in cellular redox state regulation of acute myeloid leukemia cells
Source: Mol Oncol. 2024 Oct 4;19(1):133–50. doi: 10.1002/1878-0261.13722 (PMC11705725; doi:10.1002/1878-0261.13722)

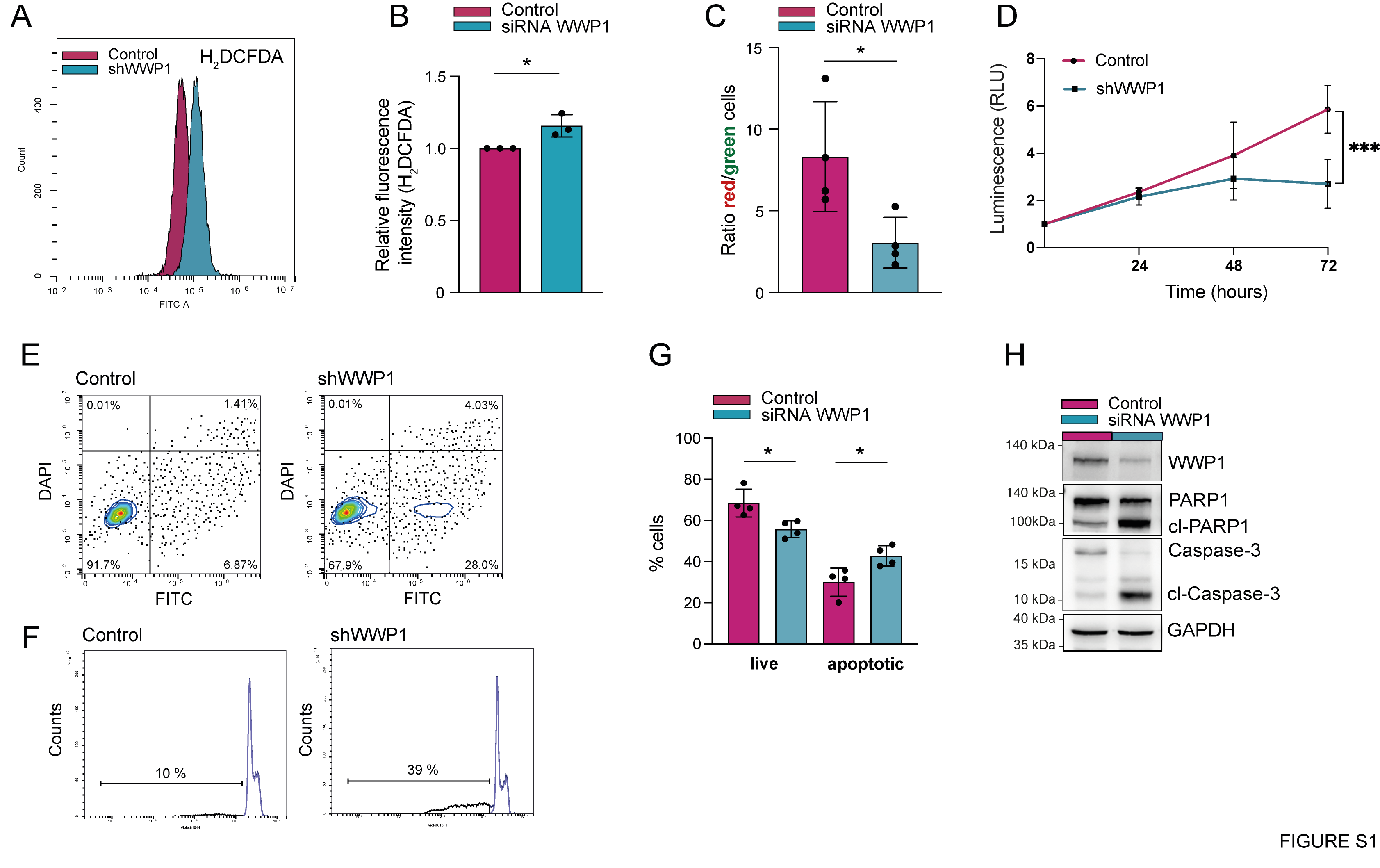

Supplement: Supplementary file 1 — Fig. S1. Oxidative stress induced by WWP1 inactivation triggers apoptotic cell death. Fig. S2. WWP1 binds TXNIP and promotes its ubiquitination independently of ERK‐mediated phosphorylation. Fig. S3. WWP1 influences TXNIP‐mediated regulation of glucose uptake and consumption. [file MOL2-19-133-s003.zip › Figure S1.tif]

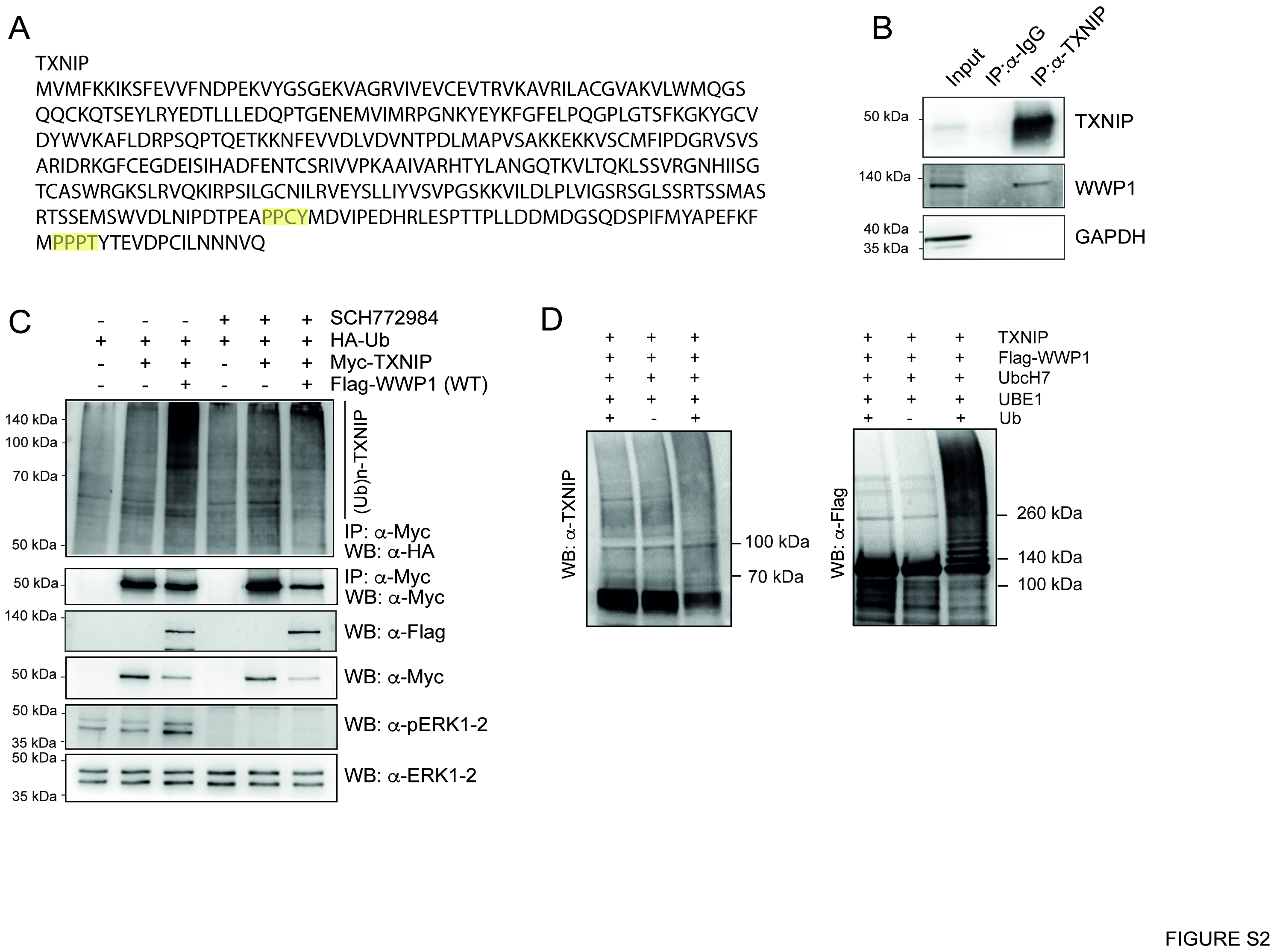

Supplement: Supplementary file 1 — Fig. S1. Oxidative stress induced by WWP1 inactivation triggers apoptotic cell death. Fig. S2. WWP1 binds TXNIP and promotes its ubiquitination independently of ERK‐mediated phosphorylation. Fig. S3. WWP1 influences TXNIP‐mediated regulation of glucose uptake and consumption. [file MOL2-19-133-s003.zip › Figure S2.tif]

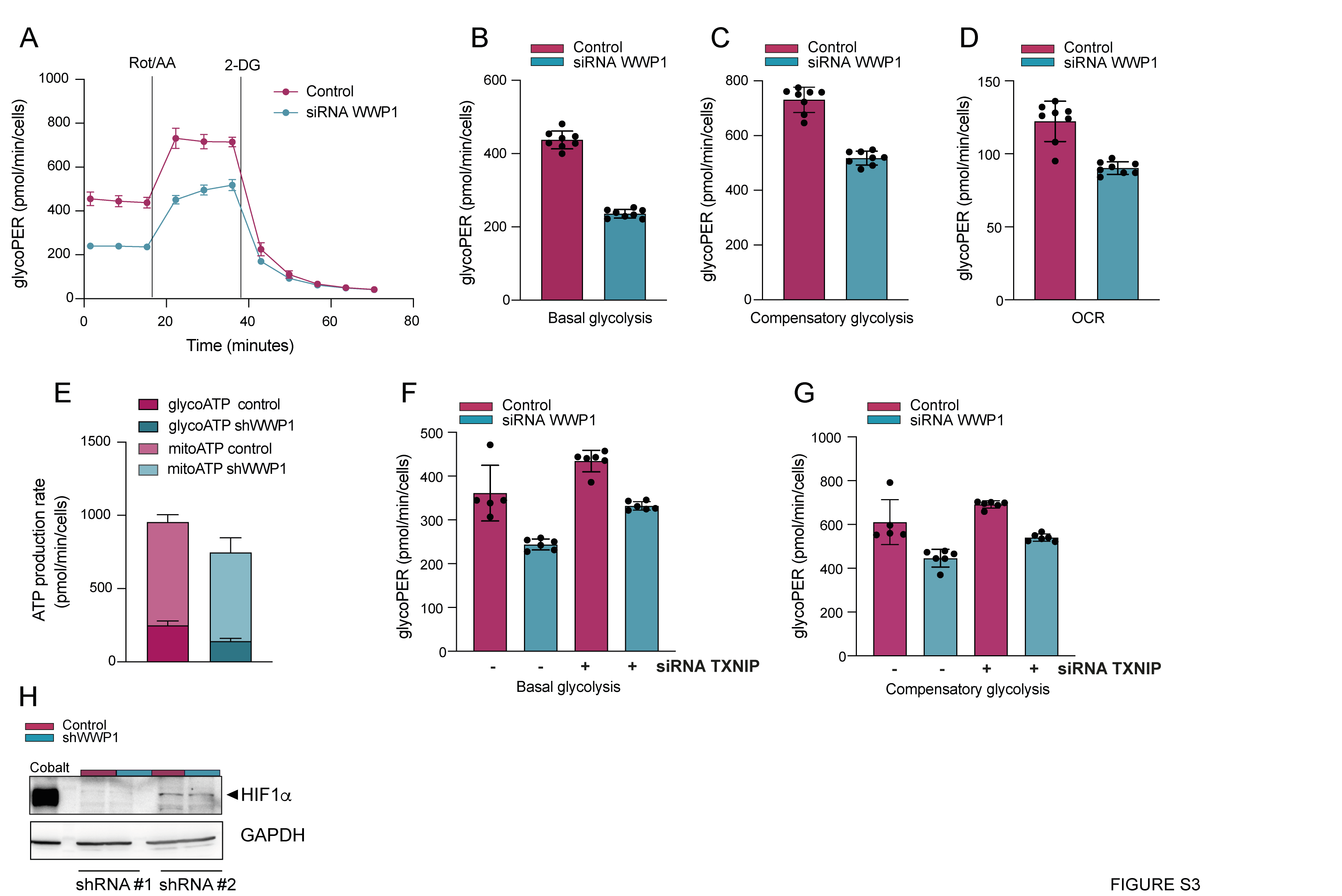

Supplement: Supplementary file 1 — Fig. S1. Oxidative stress induced by WWP1 inactivation triggers apoptotic cell death. Fig. S2. WWP1 binds TXNIP and promotes its ubiquitination independently of ERK‐mediated phosphorylation. Fig. S3. WWP1 influences TXNIP‐mediated regulation of glucose uptake and consumption. [file MOL2-19-133-s003.zip › Figure S3.tif]
