# Supplementary material for: Thioredoxin‐interacting protein (TXNIP) is a substrate of the NEDD4‐like E3 ubiquitin‐protein ligase WWP1 in cellular redox state regulation of acute myeloid leukemia cells
Source: Mol Oncol. 2024 Oct 4;19(1):133–50. doi: 10.1002/1878-0261.13722 (PMC11705725; doi:10.1002/1878-0261.13722)
Supplement: Supplementary file 2 — Table S1. List of the WWP1 interactors. [file MOL2-19-133-s001.docx]

| #node1 | node2 | node1_string_id | node2_string_id | experimentally determined interaction SCORE |
| --- | --- | --- | --- | --- |
| RPS27A | UBC | 9606,ENSP00000272317 | 9606,ENSP00000441543 | 0,955 |
| UBC | RPS27A | 9606,ENSP00000441543 | 9606,ENSP00000272317 | 0,955 |
| UBE2L3 | WWP1 | 9606,ENSP00000485133 | 9606,ENSP00000427793 | 0,847 |
| WWP1 | UBE2L3 | 9606,ENSP00000427793 | 9606,ENSP00000485133 | 0,847 |
| UBE2L3 | UBC | 9606,ENSP00000485133 | 9606,ENSP00000441543 | 0,833 |
| UBC | UBE2L3 | 9606,ENSP00000441543 | 9606,ENSP00000485133 | 0,833 |
| UBE2L3 | RPS27A | 9606,ENSP00000485133 | 9606,ENSP00000272317 | 0,829 |
| RPS27A | UBE2L3 | 9606,ENSP00000272317 | 9606,ENSP00000485133 | 0,829 |
| UBC | WWP1 | 9606,ENSP00000441543 | 9606,ENSP00000427793 | 0,659 |
| WWP1 | UBC | 9606,ENSP00000427793 | 9606,ENSP00000441543 | 0,659 |
| UBE2L3 | WWP1 | 9606,ENSP00000400906 | 9606,ENSP00000427793 | 0,606 |
| WWP1 | UBE2L3 | 9606,ENSP00000427793 | 9606,ENSP00000400906 | 0,606 |
| RPS27A | WWP1 | 9606,ENSP00000272317 | 9606,ENSP00000427793 | 0,589 |
| WWP1 | RPS27A | 9606,ENSP00000427793 | 9606,ENSP00000272317 | 0,589 |
| WBP2 | WWP1 | 9606,ENSP00000467579 | 9606,ENSP00000427793 | 0,574 |
| WWP1 | WBP2 | 9606,ENSP00000427793 | 9606,ENSP00000467579 | 0,574 |
| UBC | UBE2L3 | 9606,ENSP00000441543 | 9606,ENSP00000400906 | 0,569 |
| UBE2L3 | UBC | 9606,ENSP00000400906 | 9606,ENSP00000441543 | 0,569 |
| SMAD6 | WWP1 | 9606,ENSP00000288840 | 9606,ENSP00000427793 | 0,563 |
| SMAD7 | WWP1 | 9606,ENSP00000262158 | 9606,ENSP00000427793 | 0,563 |
| WWP1 | SMAD7 | 9606,ENSP00000427793 | 9606,ENSP00000262158 | 0,563 |
| WWP1 | SMAD6 | 9606,ENSP00000427793 | 9606,ENSP00000288840 | 0,563 |
| ARRDC1 | WWP1 | 9606,ENSP00000360475 | 9606,ENSP00000427793 | 0,556 |
| ARRDC3 | WWP1 | 9606,ENSP00000265138 | 9606,ENSP00000427793 | 0,556 |
| TXNIP | WWP1 | 9606,ENSP00000462521 | 9606,ENSP00000427793 | 0,556 |
| WWP1 | ARRDC3 | 9606,ENSP00000427793 | 9606,ENSP00000265138 | 0,556 |
| WWP1 | ARRDC1 | 9606,ENSP00000427793 | 9606,ENSP00000360475 | 0,556 |
| WWP1 | TXNIP | 9606,ENSP00000427793 | 9606,ENSP00000462521 | 0,556 |
| SMAD6 | SMAD7 | 9606,ENSP00000288840 | 9606,ENSP00000262158 | 0,483 |
| SMAD7 | SMAD6 | 9606,ENSP00000262158 | 9606,ENSP00000288840 | 0,483 |
| RPS27A | UBE2L3 | 9606,ENSP00000272317 | 9606,ENSP00000400906 | 0,475 |
| UBE2L3 | RPS27A | 9606,ENSP00000400906 | 9606,ENSP00000272317 | 0,475 |

**Table S1 List of the WWP1 interactors**
