# Supplementary material for: Thioredoxin‐interacting protein (TXNIP) is a substrate of the NEDD4‐like E3 ubiquitin‐protein ligase WWP1 in cellular redox state regulation of acute myeloid leukemia cells
Source: Mol Oncol. 2024 Oct 4;19(1):133–50. doi: 10.1002/1878-0261.13722 (PMC11705725; doi:10.1002/1878-0261.13722)
Supplement: Supplementary file 3 — Table S2. List of primers used in the study. [file MOL2-19-133-s002.docx]

| **Name** | **Sequence** |
| --- | --- |
| **LDHA**  Fw | GGATCTCCAACATGGCAGCCTT |
| **LDHA**  Rv | AGACGGCTTTCTCCCTCTTGCT |
| **LDHB**  Fw | GGACAAGTTGGTATGGCGTGTG |
| **LDHB**  Rv | AAGCTCCCATGCTGCAGATCCA |
| **TBP**  Fw | TCAAACCCAGAATTGTTCTCCTTAT |
| **TBP**  Rv | CCTGAATCCCTTTAGAATAGGGTAGA |
| **GLUT1** Fw | CTTCCTGCTCATCAACCGCAA |
| **GLUT1** Rv | ACCTTCTTCTCCCGCATCATC |
| **GLUT4** Fw | CGTCTTCCTTCTATTTGCGGT |
| **GLUT4** Rv | TGGGTTTCACCTCCTGCTCTA |
| **Kpn-MYC_1**  Fw | GGGGTACCATGGAACAGAAGCTGATCTCAGAGGAGGACCTGCTGGGATCCATGGTGATGTTCAAGAAGATCAAGTCTTTTG |
| **EcoRI-392**  Rv | CGGAATTCTCACTGCACATTGTTGTTGAGGATGC |

**Table S2 List of primers used in the study**
